# Photodeposition‐Based Synthesis of TiO_2_@IrO_x_ Core–Shell Catalyst for Proton Exchange Membrane Water Electrolysis with Low Iridium Loading

**DOI:** 10.1002/advs.202402991

**Published:** 2024-06-14

**Authors:** Darius Hoffmeister, Selina Finger, Lena Fiedler, Tien‐Ching Ma, Andreas Körner, Matej Zlatar, Birk Fritsch, Kerstin Witte Bodnar, Simon Carl, Alexander Götz, Benjamin Apeleo Zubiri, Johannes Will, Erdmann Spiecker, Serhiy Cherevko, Anna T. S. Freiberg, Karl J. J. Mayrhofer, Simon Thiele, Andreas Hutzler, Chuyen van Pham

**Affiliations:** ^1^ Forschungszentrum Jülich GmbH Helmholtz Institute Erlangen‐Nürnberg for Renewable Energy 91058 Erlangen Germany; ^2^ Department Chemical and Biological Engineering Friedrich‐Alexander‐Universität Erlangen‐Nürnberg 91058 Erlangen Germany; ^3^ Fraunhofer Institute for Microstructure of Materials and Systems (IMWS) 06120 Halle Germany; ^4^ Fraunhofer Center for Silicon Photovoltaics 06120 Halle Germany; ^5^ Institute of Micro‐ and Nanostructure Research (IMN) and Center for Nanoanalysis and Electron Microscopy (CENEM) Interdisciplinary Center for Nanostructured Films (IZNF) Friedrich‐Alexander‐Universität Erlangen‐Nürnberg 91058 Erlangen Germany

**Keywords:** core–shell catalyst, iridium utilization, low loading, proton exchange membrane water electrolysis, thickness factor

## Abstract

The widespread application of green hydrogen production technologies requires cost reduction of crucial elements. To achieve this, a viable pathway to reduce the iridium loading in proton exchange membrane water electrolysis (PEMWE) is explored. Herein, a scalable synthesis method based on a photodeposition process for a TiO_2_@IrO_x_ core–shell catalyst with a reduced iridium content as low as 40 wt.% is presented. Using this synthesis method, titania support particles homogeneously coated with a thin iridium oxide shell of only 2.1 ± 0.4 nm are obtained. The catalyst exhibits not only high ex situ activity, but also decent stability compared to commercially available catalysts. Furthermore, the unique core–shell structure provides a threefold increased electrical powder conductivity compared to structures without the shell. In addition, the low iridium content facilitates the fabrication of sufficiently thick catalyst layers at decreased iridium loadings mitigating the impact of crack formation in the catalyst layer during PEMWE operation. It is demonstrated that the novel TiO_2_@IrO_x_ core–shell catalyst clearly outperforms the commercial reference in single‐cell tests with an iridium loading below 0.3 mg_Ir_ cm^−2^ exhibiting a superior iridium‐specific power density of 17.9 kW g_Ir_^−1^ compared to 10.4 kW g_Ir_^−1^ for the commercial reference.

## Graphical abstract

A facile and scalable synthesis method for a TiO_2_@IrO_x_ core–shell electrocatalyst is developed, and its stability and superior performance for the acidic oxygen evolution reaction compared to a commercial catalyst is demonstrated in both ex situ and single‐cell electrochemical measurements.

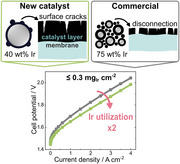

## 1 Introduction

Green hydrogen is a promising energy carrier that can bridge the gap between the supply and demand of renewable energies. Amongst the technologies to produce hydrogen by water splitting, proton exchange membrane water electrolysis (PEMWE) is one of the most promising approaches. The major advantages of this technology are its high power density resulting in a compact stack architecture, and its capability to follow fast load fluctuations.^[^ ^1^ ^]^ These two advantages are significant considering a future scale‐up of the technology to Gigawatt (GW) scales and the connection to renewable, inherently intermittent energy sources.

Due to their high activity and stability under the harsh PEMWE operation conditions (low pH and high potentials at elevated temperatures) of the acidic oxygen evolution reaction (OER), state‐of‐the‐art electrocatalysts on the anode side of PEMWE cells are usually iridium‐based. Although alternative materials such as ruthenium can show superior OER activity in acidic media compared to iridium, the higher dissolution rate of Ru under PEMWE conditions strongly constrains its long‐term stability.^[^ ^2^, ^3^ ^]^ However, the scarcity of iridium still limits the widespread and large‐scale implementation of PEMWE.^[^ ^4^, ^5^ ^]^

A scale‐up of the annual implementation of PEMWE to 10 GW using state‐of‐the‐art electrolyzers with loadings of 2 mg_Ir_ cm^−2^ would require 7.5 t of iridium.^[^ ^5^ ^]^ In contrast, the global iridium supply in 2022 only amounted to 6.8 t per year.^[^ ^6^ ^]^ Calculations considering the scarcity of iridium and the projected scale‐up to the GW scale reveal that an iridium‐specific power density of 50–100 kW g_Ir_^−1^ is required to adopt PEMWE successfully.^[^ ^7^, ^8^, ^9^ ^]^ The iridium‐specific power density in recent publications ranges between 9–16 kW g_Ir_^−1^.^[^ ^5^, ^8^, ^10^ ^]^ Thus, the iridium utilization must be ultimately enhanced for a future scale‐up of PEMWE. This is achievable only by designing electrodes with strongly reduced iridium loadings while simultaneously maintaining high PEMWE performance.

Decreasing the loading, however, usually involves a reduction of catalyst layer thickness. Importantly, it was shown that below a certain threshold of layer thickness or loading (e.g., for Umicore Elyst Ir75 0480: < 2 µm or < 0.5 mg_Ir_ cm^−2^),^[^ ^8^ ^]^ the structural integrity and, thus, the in‑plane electrical connectivity within the layer is disrupted. Additionally, cracks can form during operation in these thin catalyst layers. In combination with the comparably large pore size of the porous transport layers (PTLs), these cracks result in disconnected and therefore inactive catalyst islands. This decreased catalyst utilization comes along with significant performance losses.^[^ ^10^, ^11^, ^12^ ^]^

To overcome the problems associated with catalyst layers that are too thin, the iridium content in the catalyst powder should be decreased to enable the fabrication of sufficiently thick catalyst layers with low iridium loadings. In other words, advanced electrode architectures require a high electrode thickness factor, which is, per definition, the electrode thickness as a function of catalyst loading.^[^ ^1^, ^8^, ^13^ ^]^

Common strategies to reduce the iridium content in the catalyst powder are either to alloy iridium with more abundant elements or to support the iridium. Alloying has been shown with many different elements, such as Fe,^[^ ^14^ ^]^ Cr,^[^ ^15^ ^]^ and Ni.^[^ ^14^, ^16^ ^]^ However, the low dissolution stability of the alloying elements is a concern, considering both catalyst durability and membrane poisoning.^[^ ^17^ ^]^ Using support materials for iridium catalysts instead can introduce a high dispersion of the iridium phase, thus increasing the number of reaction sites. However, the electrical connection of the iridium sites must be ensured to render them active for the OER. To achieve this, several conductive supports have been tested, for example, antimony‐doped tin oxide (ATO)^[^ ^18^ ^]^ and tantalum‐doped TiO_2_^[^ ^19^ ^]^ showing high conductivity. But once more, the dissolution stability of these materials is questionable; illustratively, a high antimony dissolution from ATO has been shown in three‐electrode electrochemical tests.^[^ ^20^ ^]^

To avoid support degradation, TiO_2_ is established as a stable support, both in academic research^[^ ^13^, ^21^, ^22^, ^23^, ^24^, ^25^ ^]^ and in industry. Nevertheless, the electrical conductivity of TiO_2_ is so low that it is regarded as an insulator for the application in PEMWE.^[^ ^22^ ^]^ Therefore, the conductivity of TiO_2_‐supported iridium catalysts must be ensured solely by the iridium phase. A core–shell structure is usually aimed for to guarantee a percolating pathway from particle to particle.^[^ ^13^, ^21^, ^24^, ^25^, ^26^ ^]^

Here, a delicate balance must be found between reducing the iridium content on the one hand and achieving sufficient conductivity on the other hand. Generally, to ultimately decrease the iridium content, an iridium shell as thin as possible is desirable, which, however, reduces the electric conductivity. Additionally, choosing support particles with a low specific surface area can decrease the required amount of iridium because less surface needs to be covered for a closed shell, as pointed out by Bernt et al.^[^ ^13^ ^]^

In this regard, photodeposition is a suitable method to produce core–shell particles. During photodeposition synthesis, a mixture of semiconducting particles (e.g., TiO_2_) and metal ions (e.g., Ir^X+^) is exposed to electromagnetic radiation with an energy exceeding the band gap energy of the semiconductor (e.g., UV light). The irradiation generates electron–hole pairs inside the semiconductor. Usually, a sacrificial agent is introduced as a hole scavenger, enabling the electron in the conduction band to reduce metal ions on the semiconductor surface. During this process, metal ions are deposited as metal (oxide) seeds on the semiconducting particles. However, photodeposition processes are influenced by various parameters, such as the choice of precursor, hole scavenger, pH, and gas saturation, as pointed out by Wenderich et al.^[^ ^27^ ^]^ Additionally, the role of the hole scavenger is still not well understood in various photodeposition studies. Nonetheless, the photodeposition process has been found applicable in synthesizing noble metal/semiconductor compounds in different research areas, such as photocatalytic water splitting,^[^ ^28^, ^29^ ^]^ wastewater treatment,^[^ ^30^ ^]^ and air purification.^[^ ^31^ ^]^

While first successes with photodeposited iridium oxide on TiO_2_ have been reported,^[^ ^32^ ^]^ they are still scarce in the research for PEMWE. Albeit this demonstrates the general applicability of photodeposition to this research field, neither a continuous shell was achieved so far nor was the catalyst characterized on PEMWE single‐cell level.

In this work, we present TiO_2_@IrO_x_ core–shell particles with a homogeneous, nanometer‐thin iridium‐shell as OER electrocatalyst that is synthesized by a facile and scalable photodeposition process. Combining this novel synthesis method with low surface area TiO_2_ support particles enables the synthesis of catalysts with reduced iridium contents down to only 40 wt.% iridium. Herein, we first extensively analyze the novel synthesis method using transmission electron microscopy (TEM), X‐ray photoelectron spectroscopy (XPS), and nuclear magnetic resonance (NMR) spectroscopy to shed light on the shell formation mechanism. Moreover, the final catalyst is compared to commercially available catalysts in both three‐electrode setups and single‐cell testing in a catalyst‐coated membrane (CCM) configuration regarding performance and stability. We demonstrate that our catalyst layer architecture outperforms state‐of‐the‐art catalyst layers at low loadings, approaching the power density target of the U.S. Department of Energy (DOE) for 2026 of 10 kW g_PGM_^−1^. The stability of the catalyst was tested using a scanning flow cell setup coupled on‐line to an inductively coupled plasma mass spectrometer (SFC‐ICP‐MS) as well as in single‐cell measurements, indicating durability, which is on par with modern, iridium‐based OER catalysts. Ultimately, we emphasize that the presented synthesis method is readily scalable, as large UV reactors for water purification with throughputs of >1000 m^3^ per day are already implemented.^[^ ^33^ ^]^

## 2 Results and Discussion

### 2.1 Synthesis and Physical Characterization of TiO_2_@IrO_x_ Core–Shell Particles

**Figure 1:**
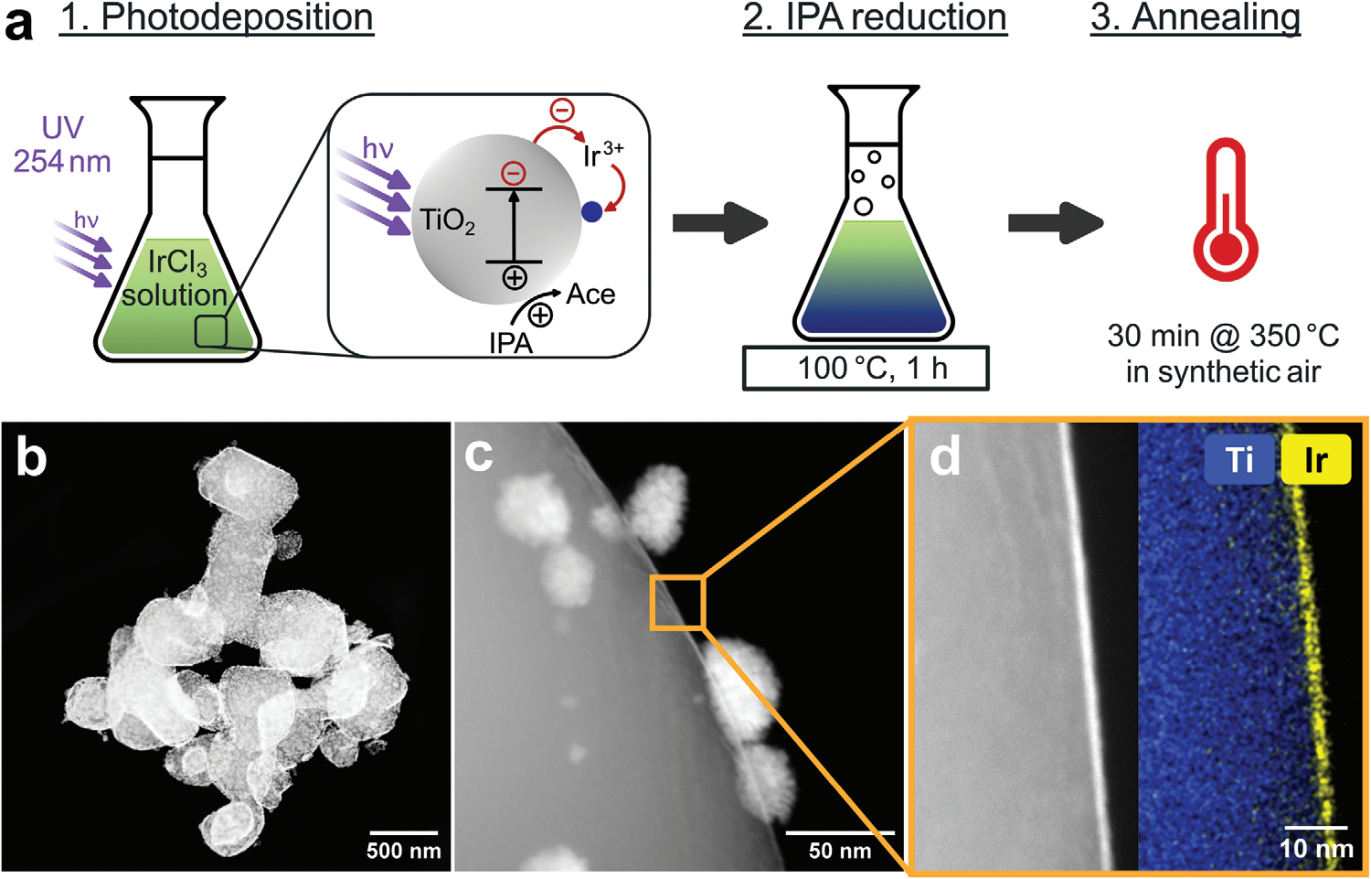
a) Schematics of three‐step synthesis based on the oxidation of isopropanol (IPA) to acetone (Ace), and b–d) HAADF‐STEM/EDX spectrum images of the synthesized TiO_2_@IrO_x_ core–shell catalyst.

Herein, the synthesis route of the TiO_2_@IrO_x_ core–shell particles comprises three individual steps, which are schematically depicted in **Figure** **1**a.

The first step is a photodeposition process exploiting the semiconducting properties of the TiO_2_ support particles: By using photons with energy exceeding that of the semiconductor's bandgap (*E*_g_ (TiO_2_) = 3.0–3.2 eV),^[^ ^34^, ^35^ ^]^ electrons inside the semiconductor can be excited from the valence to the conduction band. Usually, the electron–hole pairs would recombine and dissipate heat. However, if a hole scavenger is introduced, it is readily oxidized by the hole in the semiconductor so that an electron remains in the conduction band of the semiconductor. This electron is available for reactions on the surface of the TiO_2_ particles. In this work, isopropanol was chosen as a hole scavenger.

Oxidation of isopropanol yields acetone, while Ir^3+^ is reduced to metallic Ir^0^ as schematically shown in Figure 1a. This process is known as reductive photodeposition.^[^ ^27^ ^]^ Alternatively, oxidative photodeposition for iridium has also been reported with direct deposition of IrO_2_.^[^ ^32^ ^]^ The photodeposition mechanism in this work is described in detail below.

To utilize all of the iridium precursor, (IrCl3) the second synthesis step employs the remaining isopropanol as a reduction agent at 100 °C. This approach was chosen since it requires no additional chemicals, rendering the synthesis method easily scalable.

As a third step, the catalyst is annealed at 350 °C under oxidative conditions. This temperature was chosen as a compromise between activity and stability, which is a well‐known trade‐off for iridium‐based catalysts.^[^ ^20^, ^36^, ^37^ ^]^

The TiO_2_@IrO_x_ core–shell particles synthesized via this three‐step method are shown in HAADF‐STEM images in Figure 1b–d. The core–shell catalyst is based on a TiO_2_ support with a comparably large particle size of 750 ± 360 nm. Two different iridium structures make up the active catalyst: A 2.1 ± 0.4 nm thin and homogeneous shell of iridium phase (iridium oxide, as evidenced below) covers the whole TiO_2_ support particle, and on top of this shell, nanoparticles with a sponge‐like morphology and a size of 34 ± 9 nm are formed. The thin shell is essential because it forms an electrically percolating pathway that connects the dispersed iridium nanoparticles with each other and with the porous transport layer (PTL). In other words, this thin IrO_x_ shell enables conductive TiO_2_@IrO_x_ catalysts with low iridium contents. On the other hand, the additional iridium nanoparticles can benefit the catalyst by introducing a high surface area for the catalytic water splitting.

The rationale behind choosing TiO_2_ particles with a large diameter is their low specific surface area, which allows for the full iridium‐covered core–shell particles at a low iridium content. For the TiO_2_ particles used here, a BET surface of 3.0 m^2^ g^−1^ was measured. Assuming a 2.1 nm thin shell of iridium oxide, only 6 wt.% iridium in the catalyst powder is needed to complete the core–shell structure (for this calculation, *ρ* (IrO_2_) = 11.66 g cm^−3^ was used). The measured iridium content after the photodeposition is only 2.7 ± 0.6 wt.% as determined via XRF measurements, equating to an average shell thickness of ≈1 nm. This discrepancy in iridium content and the observed shell thickness should be further investigated. In this work, an iridium content of 40 wt.% was chosen to guarantee an excess of iridium for the shell formation and additional active sites of the nanoparticles. Experimentally, this iridium content was confirmed to be 39.8 ± 2.5 wt.%. This amount is still much lower than the iridium content in commercial catalysts which is usually between 75 and 100 wt.% (e.g., Umicore Elyst Ir75 or iridium black).

**Figure 2:**
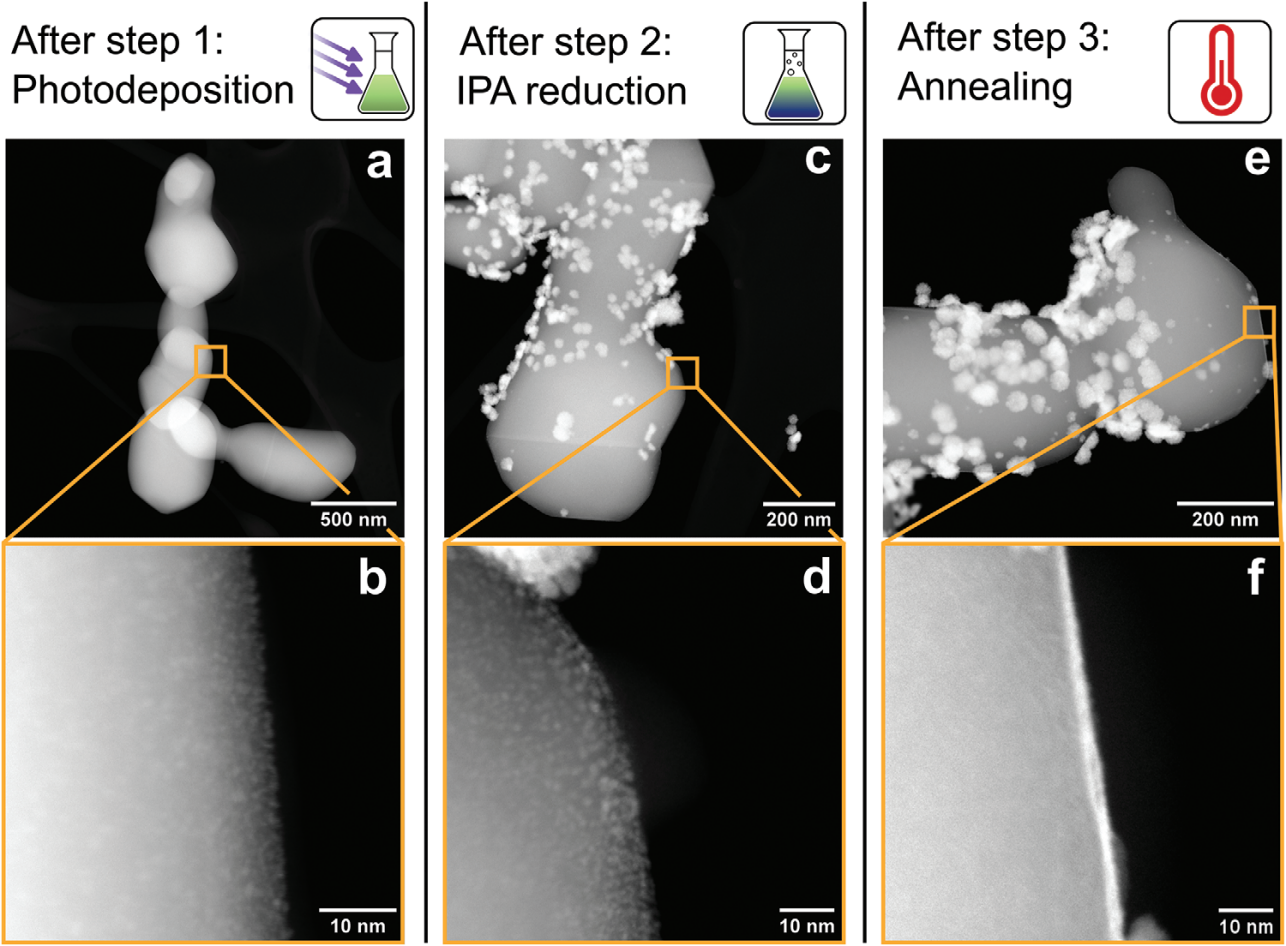
HAADF‐STEM analysis of the TiO_2_@IrO_x_ catalyst after each synthesis step. The catalyst after a, b) photodeposition, c, d) photodeposition and IPA reduction, and e, f) photodeposition, IPA reduction, and annealing.

To gain insights into the formation mechanisms of the different catalyst structures, the particles were analyzed after each individual synthesis step by HAADF‐STEM and EDX spectrum imaging. As shown in **Figure** **2**a, there is no formation of larger (>10 nm) iridium structures after the photodeposition step. However, looking at the surface of the TiO_2_ support particles reveals the existence of small iridium seeds with an average size of 0.9 ± 0.2 nm (Figure 2b). We conclude that these seeds are the result of the photodeposition. The participation of isopropanol as a hole scavenger in the photodeposition was evidenced by detecting its oxidized form (acetone) in the ^1^H NMR spectrum of the reaction filtrate (Figure S2a,b, Supporting Information). We hypothesize that the photodeposition process is self‐limiting because Ir/TiO_2_ compounds are known as effective catalysts for photoelectrochemical water splitting,^[^ ^38^, ^39^ ^]^ which might compete with the photodeposition process. Moreover, the formed iridium particle layer could hinder the UV light from entering the TiO_2_ particles.

The second synthesis step uses isopropanol for the chemical reduction of the iridium ions, which only a few other studies have reported.^[^ ^40^, ^41^ ^]^ Again, the reaction pathway was confirmed by detecting an increased amount of acetone in the ^1^H NMR spectrum of the filtrate after the second reaction step (Figure S2c, Supporting Information). During this second step, iridium nanoparticles with an average size of 34 ± 9 nm have formed on top of the seeds from the first reaction step (Figure 2c).

It should be noted that some unsupported iridium particle clusters were observed. However, as the measured iridium content is very close to the calculated value (as described above), we conclude that the majority of the particles formed on the support particles. On the surface of the TiO_2_ support, the iridium seeds from the first synthesis step increase in size to 1.4 ± 0.4 nm but generally maintain their morphology (Figure 2d). After the third synthesis step, the iridium nanoparticles retained their size and morphology (Figure 2e). Notably, a continuous shell of iridium oxide on the TiO_2_ core has formed (Figure 2f; more HAADF‐STEM and EDX spectrum images are shown in Figure S3, Supporting Information). Therefore, we conclude that the closed shell is formed during the annealing step.

To verify that the shell forms from the iridium seeds previously grown by photodeposition, we performed the synthesis route as described but without the second synthesis step (isopropanol reduction). As shown in Figure S4 (Supporting Information), a continuous IrO_x_ shell is also observed for these particles that only underwent photodeposition and annealing. Although the formation of the shell can thus be clearly attributed to these two synthesis steps, the exact mechanism behind the shell formation has yet to be elucidated. A possible explanation could be the fulfillment of a wetting condition during the annealing step.

As an additional validation step of the synthesis, another batch of particles was synthesized with an omitted photodeposition step. The iridium content of this catalyst is measured to be 38 ± 3 wt.%, which is similar to the batch that underwent all synthesis steps. These particles, which only underwent isopropanol reduction and annealing, do not exhibit a shell, as shown in Figure S5 (Supporting Information). This observation emphasizes the importance of the photodeposition step in forming the core–shell structure.

In general, a high specific surface area is important for catalysts to provide as many catalytic sites as possible. The final core–shell catalyst particles exhibit a BET surface area of 9.4 ± 0.1 m^2^ g^−1^ which is three times larger than the specific surface area of the bare support particles. This increase in surface area is attributed to the additional iridium nanoparticles that form during the isopropanol reduction step. Although the BET surface of the TiO_2_ support was very low with only 3 m^2^ g^−1^, according to our calculation, the iridium‐specific surface area of the core–shell catalyst is ≈24 m^2^ g_Ir_^−1^, only slightly lower than that of the commercial catalysts (≈33–35 m^2^ g_Ir_^−1^; BET surface area values are shown in Figure S6, Supporting Information). We emphasize that this finding justifies using low surface area supports for OER catalysts since the iridium structures contribute the necessary surface area.

**Figure 3:**
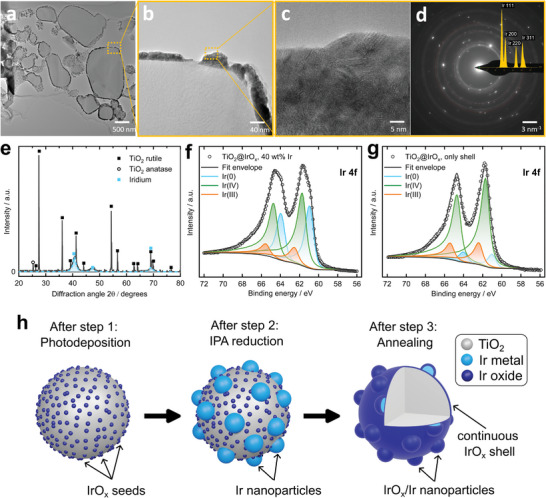
a–c) HRTEM images of a focused ion beam (FIB) lift‐out lamella of a catalyst layer incorporating the TiO_2_@IrO_x_ core–shell catalyst. d) ED pattern from the lamella sample (yellow overlay: expected rotationally averaged peaks of arbitrarily oriented fcc Ir). e) XRD spectrum of the TiO_2_@IrO_x_ core–shell catalyst. f, g) Ir 4f XPS spectra and deconvolution fittings of the TiO_2_@IrO_x_ core–shell catalyst and the catalyst with only the thin shell (only photodeposition and annealing), respectively. h) Schematic illustration summarizing the morphology and oxidation state after every synthesis step.

After clarifying the origins of the different catalyst features, we now investigate the morphology and oxidation state of the novel core–shell catalyst. For this purpose, bright field (HR)TEM and ED analyses were performed on a thin catalyst layer lamella prepared by FIB‐SEM lift out from a CCM and subsequent FIB milling to ≈100 nm thickness. As shown in **Figure** **3**a–c, the additional iridium nanoparticles are evenly distributed around the TiO_2_ support. Notably, in contrast to the HAADF‐STEM depicted above, bright field (HR)TEM is employed here. Consequently, the iridium phase appears now much darker than the TiO_2_ support. While the inter‐particle electrical percolation in the catalyst layer (CL) in Figure 3a seems imperfect, it should be kept in mind that the FIB lamella only shows a thin slice of the CL, and percolation might exist in the third dimension. A small gap in the shell is observed in Figure 3b, which could be attributed to an incomplete coverage during synthesis or dewetting during FIB lamella preparation or during imaging, as we witnessed beam‐induced dewetting of the iridium shell at high electron doses. A closer inspection of the iridium layer at higher magnification reveals that lattice fringes of metallic iridium are present, corresponding to their larger scale equivalent measured by ED (Figure 3d). Moreover, HRTEM (Figure 3c) shows that the Ir phase consists of many randomly oriented small crystalline grains in the 3–8 nm range. This is confirmed by a Rietveld refinement analysis of the XRD measurement (Figure 3e) that yields an average crystallite size of 6.2 ± 0.2 nm for the crystalline iridium metal phase still present after annealing.

The composition of the support based on XRD shows mainly the rutile structure of TiO_2_ with small amounts of anatase. Interestingly, the iridium phase appears mainly metallic, as both ED patterns (Figure 3d; Figure S7, Supporting Information) and XRD measurements (Figure 3e) reveal. Although no complete oxidation can be expected from an annealing temperature of 350 °C,^[^ ^25^, ^42^ ^]^ it is interesting to note that crystalline IrO_2_ could not be detected. Nonetheless, some degree of oxidation is expected, and an amorphous IrO_x_ phase is likely developed on the surface of the iridium nanoparticles that cannot be detected in our diffraction measurements. To further investigate the oxidation state of the iridium, XPS measurements were performed.

Figure 3f shows the XPS Ir 4f spectrum of the synthesized TiO_2_@IrO_x_ catalyst. The deconvolution of the peaks reveals a surface with a mixed iridium oxidation state of Ir(0), Ir(III), and Ir(IV) present in the order of Ir(IV) > Ir(0) > Ir(III) (values shown in Table S2, Supporting Information). Combining this finding about the surface composition with the detected metallic crystalline iridium in XRD measurements suggests that the dispersed nanoparticles consist of an IrO_x_/Ir structure with a metallic core and an amorphous and substoichiometric IrO_x_ surface.

To explicitly investigate the oxidation states of the photodeposited seeds and the thin shell, additional XPS analyses were performed on samples after only photodeposition (cf. HAADF‐STEM image in Figure 2a,b) and photodeposition followed by annealing (cf. HAADF‐STEM image in Figure S3, Supporting Information), respectively. In Figure S8 (Supporting Information), the XPS spectrum of the photodeposited seeds is shown, and the presence of mostly Ir(III) and Ir(IV) species is revealed. This contrasts the detected acetone in the reaction solution, which suggests the reduction of the iridium precursor to Ir(0), as discussed above.

A possible explanation for the presence of Ir(III) and Ir(IV) species in the seed particles could lie in the anodic oxidization of Ir(0) during the concurrent water photooxidation.^[^ ^32^ ^]^ This mechanism would be analogous to the formation of a hydrous iridium oxide surface during electrochemical water splitting, which is reported in the literature for metallic iridium catalysts.^[^ ^43^, ^44^ ^]^

The XPS spectrum of only the thin IrO_x_ shell on the support is shown in Figure 3f and exhibits a high fraction of Ir(IV) species pointing toward a high oxidation state of the shell. As the Ir(IV) species were not detected as crystalline IrO_2_, and also Ir(0) and Ir(III) are present, we regard the phase of the shell as amorphous IrO_x_ with *x* being close to 2. All our findings about the synthesis steps, morphologies, and oxidation states are formulated in the schematic illustration in Figure 3h.

### 2.2 Electrochemical Characterization of Catalyst Powder

**Figure 4:**
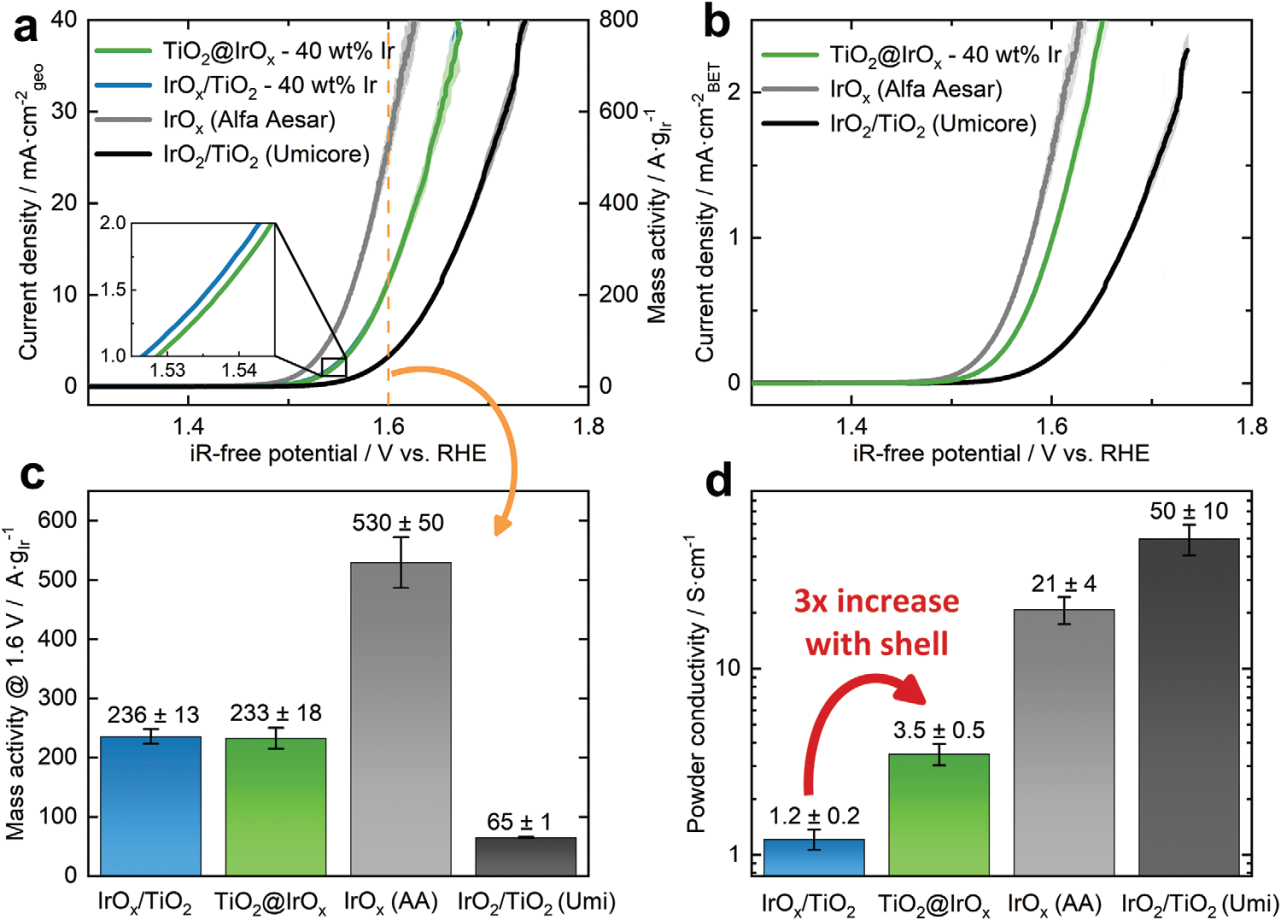
a) Geometric and b) surface‐specific LSVs and c) mass activity of the TiO_2_@IrO_x_ core–shell catalyst, the IrO_x_/TiO_2_ catalyst (no shell), and the two commercial reference catalysts, Alfa Aesar Premion and Umicore Elyst Ir75 0480. Shaded areas in the LSVs correspond to the standard deviation of three separate measurements. LSV and activity measurements were conducted in an RDE setup in Ar‐purged 0.1 m HClO_4_ at a loading of 50 µg_Ir_ cm^−2^. d) Powder conductivities of all catalysts.

The activity of the TiO_2_@IrO_x_ core–shell catalyst was analyzed in rotating disk electrode (RDE) measurements and compared to two commercial reference catalysts, IrO_x_ (Alfa Aesar Premion) and IrO_2_/TiO_2_ (Umicore Elyst Ir75 0480). Additionally, for a detailed investigation of the core–shell structure, we tested the novel catalyst without the thin shell (cf. Figure S5, Supporting Information), labeled as IrO_x_/TiO_2_. The results are shown in **Figure** **4**a. The activity of the core–shell catalyst is in between the two reference catalysts, with the Alfa Aesar catalyst showing the highest and the Umicore catalyst showing the lowest activity. The difference in activity of the Alfa Aesar and the Umicore catalyst is a well‐known behavior reported in the literature.^[^ ^21^ ^]^ It can be explained by a substoichiometric IrO_x_ phase for the Alfa Aesar catalyst, which results in increased activity.^[^ ^45^ ^]^ The fully oxidized rutile IrO_2_ phase of the Umicore catalyst,^[^ ^46^ ^]^ on the contrary, is highly crystalline and yields lower activities.

Interestingly, the activity of the novel catalyst with and without a shell is almost identical. To investigate the contributions of the thin IrO_x_ shell and the additional IrO_x_/Ir nanoparticles to the OER activity, we measured the mass activity of the TiO_2_@IrO_x_ core–shell catalyst with only the shell (no additional nanoparticles). As shown in Figure S9 (Supporting Information), it exhibits a similar mass activity (within large error bars) as the catalyst with the additional nanoparticles, pointing toward an equal contribution of the IrO_x_ shell and the IrO_x_/Ir nanoparticles particles to the overall mass activity.

Although this result may suggest that the thin shell has no advantage, we emphasize that the conductivity of the catalyst only plays a minor role in RDE measurements due to the thin catalyst layers and the highly conductive electrodes.^[^ ^47^ ^]^

To further investigate the surface‐specific activity of the core–shell catalyst, RDE results were normalized to the BET surface area (BET surface area values are shown in Figure S6, Supporting Information). For this analysis, we assumed that all the TiO_2_ support is covered with iridium oxide so that the BET analysis equates to the active catalyst surface area. Thus, this analysis is not possible for the IrO_x_/TiO_2_ catalyst (without a closed shell). As shown in Figure 4b, the surface‐specific activity of the core–shell catalyst still lies between the two commercial references. However, it is even closer to the activity of the Alfa Aesar catalyst, indicating that the activity per catalytic site is relatively high. This finding is further supported by similar Tafel slopes for the core–shell catalyst and the Alfa Aesar catalyst, with 47 and 48 mV dec^−1^. In contrast, the Umicore catalyst exhibits a higher Tafel slope of 56 mV dec^−1^ (Figure S10, Supporting Information). We explain this high surface‐specific activity of the core–shell catalyst by the incomplete oxidation of the metallic iridium nanoparticles formed during the IPA reduction and the substoichiometric IrO_x_ surface phase as evidenced by physical characterization.

To approximate the electrochemically active surface area (ECSA) of the TiO_2_@IrO_x_ and the IrO_x_/TiO_2_ catalysts, pseudocapacitances of both were measured and are shown in Figure S11 (Supporting Information). This analysis reveals a higher ECSA for the core–shell specimen, which can possibly be attributed to the surface of the IrO_x_ shell itself or to an improved percolation due to the shell contacting the additional nanoparticles.

In the end, the iridium‐mass activity of the catalyst depicts the most important descriptor for the activity regarding the scarcity of iridium. The mass activity of the different catalysts is evaluated by determining the mass‐specific current densities at 1.6 V versus RHE, which are given in Figure 4c. The core–shell catalyst exhibits a moderately high mass activity of 233 ± 18 A g_Ir_^−1^, which lies in between the two commercial references with mass activities of 530 ± 50 and 65 ± 1 A g_Ir_^−1^ for the Alfa Aesar and the Umicore catalyst, respectively. For comparison to the literature, the mass activities of recently developed iridium‐based catalysts are summarized in Table S3 (Supporting Information). Noticeably, the synthesis method for the TiO_2_@IrO_x_ core–shell catalyst shows a high reproducibility with the same OER activities for three different synthesis batches, as shown in the linear sweep voltammograms (LSVs) in Figure S12 (Supporting Information).

It has been shown that the conductivity of the anodic catalyst layer is essential in PEMWE, particularly the in‐plane conductivity, which is needed to contact catalyst particles that are not in direct contact with the PTL.^[^ ^10^, ^13^ ^]^ For this reason, the powder conductivities of the catalysts were measured, and the results are presented in Figure 4d. The Alfa Aesar and Umicore commercial catalysts exhibit conductivities of 20.8 ± 3.5 and 50.0 ± 9.4 S cm^−1^, respectively. The TiO_2_@IrO_x_ core–shell catalyst exhibits a lower powder conductivity of 3.5 ± 0.5 S cm^−1^, which is attributed to the lower iridium content and the incomplete oxidation of the core–shell catalyst. Again, the high crystallinity comes into play for the Umicore catalyst, resulting in a higher conductivity than for the Alfa Aesar catalyst. The powder conductivity of ≈50 S cm^−1^ for the Umicore catalyst is in good agreement with the literature values.^[^ ^18^ ^]^ However, the same group reported a higher conductivity (416 S cm^−1^),^[^ ^13^ ^]^ which is attributed to a higher powder compression force. Thus, we emphasize the importance of measuring reference samples during powder conductivity testing of novel catalysts. Although the conductivity of the novel core–shell catalyst is comparably low, the conductivity increases by a factor of three when the catalyst undergoes the photodeposition step. This significant increase in conductivity can thus be directly attributed to the formation of the IrO_x_ shell. We emphasize that synthesizing a thin and homogeneous IrO_x_ shell paves the way toward producing OER catalysts with low iridium contents while maintaining sufficient conductivity.

### 2.3 Characterization of Catalyst Layer Structure and Conductivity

**Figure 5:**
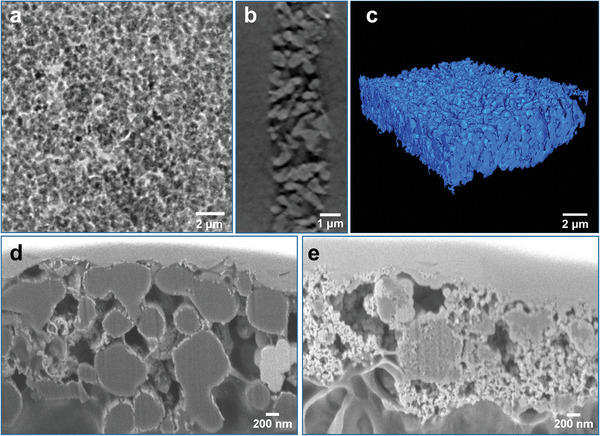
a–c) Nano‐CT measurement of an anode catalyst layer with TiO_2_@IrO_x_ core–shell particles (loading: 0.2 mg_Ir_ cm^−2^): a) Single X‐ray microscopy image (top view) from the tomographic tilt series (see Video S1 for the complete animated tilt series, Supporting Information), b) Single virtual cross‐sectional slice from the 3D reconstruction (see Video S2 for all slices, Supporting Information) and c) 3D surface rendering of the complete reconstructed volume (see Video S3 for rotation of volume, Supporting Information). d, e) FIB‐SEM images of anode CL with d) 0.2 mg_Ir_ cm^−2^ TiO_2_@IrO_x_ core–shell and e) 0.3 mg_Ir_ cm^−2^ IrO_2_/TiO_2_ (Umicore).

To test the novel core–shell structure in a PEMWE single cell, CLs were fabricated containing the TiO_2_@IrO_x_ particles. First, the CLs were analyzed regarding structure and conductivity to understand the single‐cell results better. The nano‐CT measurement of a CL incorporating the TiO_2_@IrO_x_ core–shell particles at a low loading of 0.2 mg_Ir_ cm^−2^ is displayed in **Figure** **5**a–c. The nano‐CT tilt‐series (Figure 5a; Video S1, Supporting Information) and reconstructed slices (Figure 5b; Video S2, Supporting Information) show a homogeneous layer structure with interconnection of the particles throughout the layer. Thus, electrical percolation through the CL should be enabled even at this low loading. For the reconstructed volume in Figure 5c, a catalyst volume fraction in the CL of 31.4 vol% was determined (see Figure S13 for segmentation of the volume, Supporting Information). From the ink formulation, the volume fraction of the wet ionomer is calculated to be 35.1 vol%.^[^ ^48^ ^]^ Based on this, the void volume fraction amounts to 33.5 vol%. In literature, an optimal value for void and ionomer fraction of ≈35 vol% each was found for a good balance between mass transport, and ionic and electrical conductivity.^[^ ^48^ ^]^ The highly porous structure of the TiO_2_@IrO_x_ CL can also be seen from the FIB‐SEM cross‐sectional image in Figure 5d.

In the following, the Umicore IrO_2_/TiO_2_ catalyst is chosen as a reference since it is, with regards to structure, closer to the core–shell catalyst as both are supported catalysts. The FIB‐SEM cross‐sectional image of a CL with the Umicore IrO_2_/TiO_2_ catalyst is shown in Figure 5e. When comparing Figure 5d,e, it becomes clear that the layer morphology differs strongly due to the difference in support particle size and iridium content between the two catalysts.

**Figure 6:**
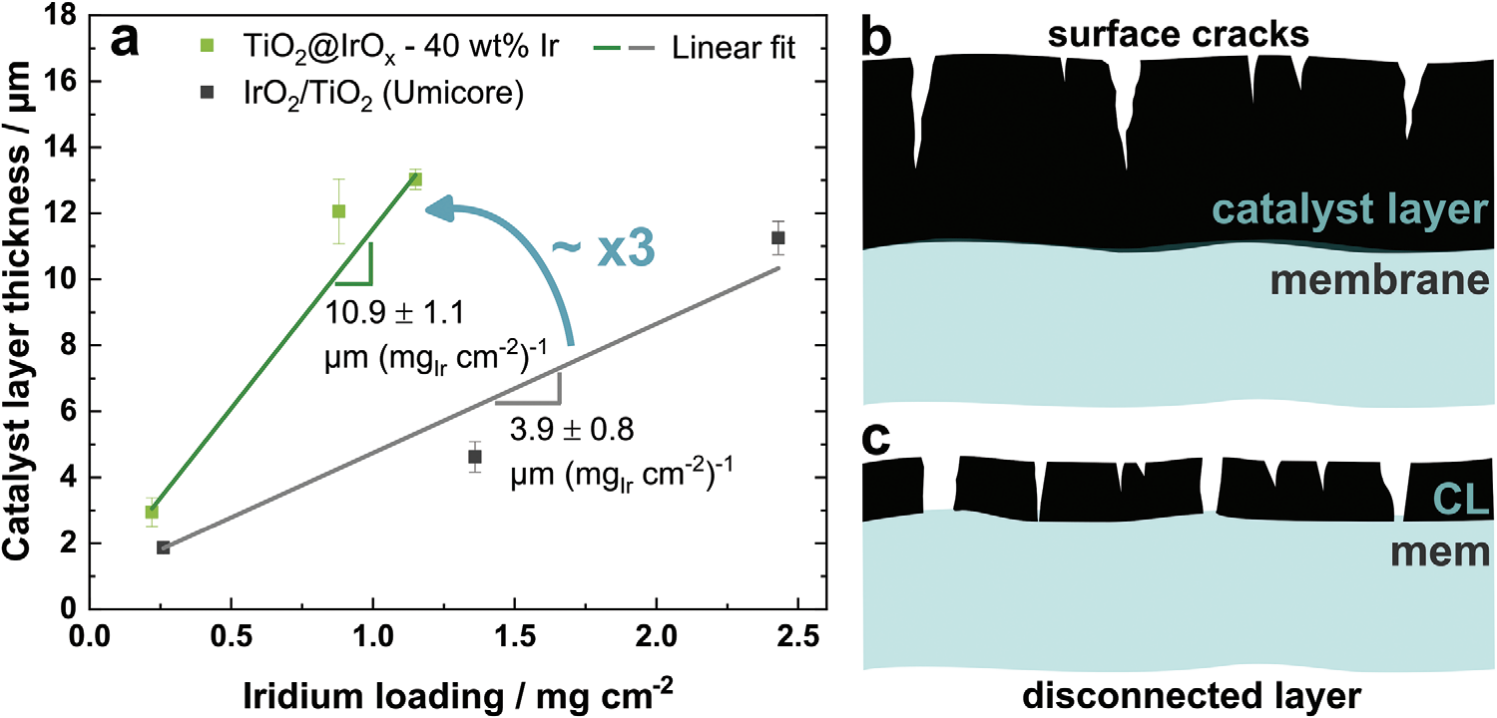
a) Anode catalyst layer thickness from SEM imaging of embedded CCMs with varying loadings. The graph shows mean values from five spots on each electrode cross‐section, including standard deviation. b, c) Schematic of catalyst layer with b) high and c) low thickness factor.

A difference in CL thickness was determined by SEM imaging of embedded CCM samples for differently loaded anodes with the core–shell and Umicore catalyst shown in **Figure** **6**a. As the slope of the graph in Figure 6a, we derived a thickness factor defined as the CL thickness normalized by the iridium loading. The thickness factor of the core–shell CL of 10.9 ± 1.1 µm (mg_Ir_ cm^−2^)^−1^ is almost three times higher than for the Umicore CL with only 3.9 ± 0.8 µm (mg_Ir_ cm^−2^)^−1^. The latter value is in good agreement with a literature value of 4.3 ± 0.3 µm (mg_Ir_ cm^−2^)^−1^ for the same catalyst material and CL fabrication method.^[^ ^8^ ^]^ As further reference, a CL fabricated via decal transfer with the IrO_x_ catalyst from Alfa Aesar yields a thickness factor of 4–5 µm (mg_Ir_ cm^−2^)^−1^.^[^ ^11^ ^]^ The difference in thickness factor mainly originates from the difference in iridium content in the catalyst powder (core–shell: 40 wt.% vs Umicore: 75 wt.% and Alfa Aesar: 86 wt.% Ir). A higher porosity due to the particle size can further contribute to this effect.

As discussed above, a higher thickness factor is necessary to reach the goal of decreasing the amount of iridium used.^[^ ^8^, ^24^ ^]^ With a higher thickness factor, thicker layers at reduced loadings can be realized compared to a catalyst with a lower thickness factor (e.g., the Umicore catalyst). When swelling of the ionomer and membrane upon water contact causes layer cracking during PEMWE operation, a thicker CL is assumed to avoid a disconnection of the layer, preventing the deactivation of parts of the catalyst and an increased electric in‐plane resistance.^[^ ^5^, ^8^ ^]^ This effect is schematically depicted in Figure 6b,c for a high‐ and low‐thickness‐factor catalyst, respectively.

A cracking of CLs was visualized in literature after contact with water at 80 °C and was shown to be more pronounced for thinner layers.^[^ ^11^ ^]^ For the extreme case of reaching full decarbonization of the transportation sector with hydrogen produced by PEMWE, calculations suggest that the iridium loading needs to be decreased to 0.05 mg_Ir_ cm^−2^ while a thickness of > 2 µm is maintained (optimal thickness ≈ 4–8 µm for Umicore Elyst Ir75).^[^ ^8^ ^]^ It becomes clear that this goal cannot be reached with unsupported (e.g., Alfa Aesar Premion) or high Ir‐content catalysts due to the low thickness factor.

With our core–shell catalyst, the thickness factor is almost tripled, bringing us closer to this goal. To fall below the threshold of 2 µm for the CL with our catalyst, the loading must be decreased below 0.18 mg_Ir_ cm^−2^. Admittedly, for the core–shell structures at loadings decreased below 0.37 mg_Ir_ cm^−2^, the layer is thinner than the optimum mentioned above of 4 µm. However, the optimal layer thickness needs to be re‐evaluated for our material since, compared to the reference material, the layer morphology differs due to a larger particle size. Moreover, due to the core–shell structure and decreased iridium content, the electrical layer connection is changed compared to the Umicore CL. Thus, in our future work, we aim to further reduce the iridium content in the catalyst powder to achieve even lower loadings at maintained layer thickness.

Another positive effect of thicker layers is the higher robustness when handling catalyst layers. This is particularly important for industrial applications, where large electrodes are used. Furthermore, we faced an issue with the Umicore catalyst: Although using the Mayer rod with the lowest wet‐film thickness, we could not reach loadings below 0.3 mg_Ir_ cm^−2^. Literature reports loadings down to 0.026 mg_Ir_ cm^−2^ with good performance (1.87 V @ 2 A cm^−2^).^[^ ^49^ ^]^ Nevertheless, CCMs with such low loadings are usually fabricated via spray coating, which is less suitable for a scale‐up to an industrial level. In contrast, the CCM fabrication used herein (Mayer rod coating and decal transfer) is closer to the slot‐die coating as a truly scalable method.

The in‐plane conductivities of pristine and dry CLs were measured for the core–shell and the Umicore catalyst. The results are shown as a function of varying iridium loadings in Figure S14 (Supporting Information). As aforementioned, the in‐plane conductivity does not play a prominent role in RDE measurements due to thin catalyst layers coated onto a highly conductive gold electrode, so these experiments cannot show an effect. In single‐cell measurements, however, a high in‐plane conductivity is important to ensure good catalyst utilization.

To assess if the connection of the layer is maintained when decreasing the loading, the in‐plane conductivity is plotted dependent on the iridium loading in Figure S14 (Supporting Information). A drop in in‐plane conductivity would be expected if the layer integrity were not maintained. This is not the case for any of the catalysts. We emphasize that the measurements are performed in dry conditions. A layer cracking is expected to occur upon membrane and ionomer swelling when they are in contact with water. Insights into the performance of CCMs in a wet state are provided by the single‐cell testing in the following section.

### 2.4 Electrochemical Characterization in PEMWE Single Cells

**Figure 7:**
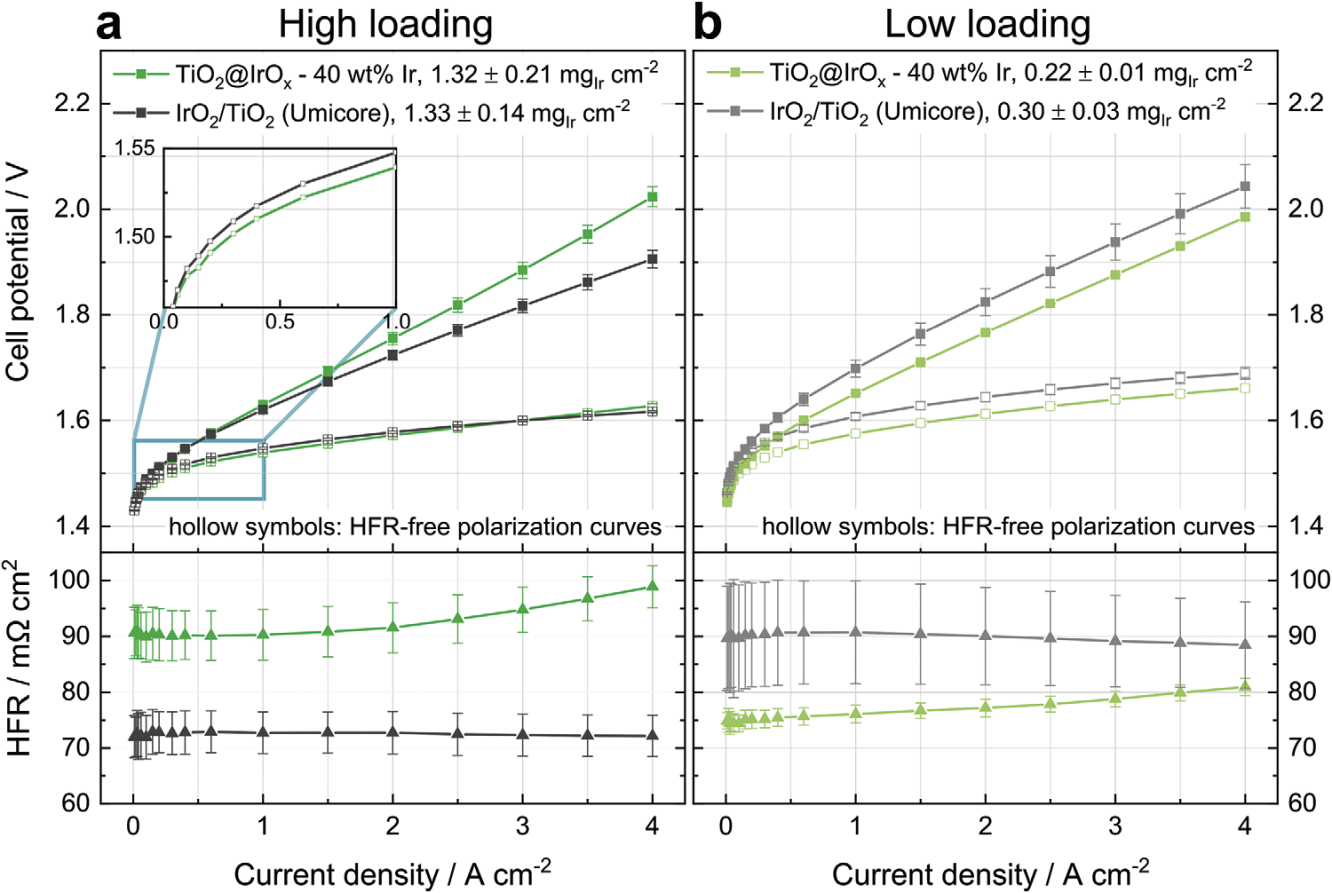
Polarization curves and HFR‐values from single‐cell testing at 80 °C and ambient pressure of TiO_2_@IrO_x_ core–shell and IrO_2_/TiO_2_ Umicore as anode catalyst at a) high and b) low loading. Inset in (a) shows a close‐up of the activation area of the HFR‐free polarization curves. A platinum‐coated Ti‐fiber PTL on the anode, a Nafion NR212 membrane (≈51 µm), and a cathode loading of 0.19 ± 0.08 mg_Pt_ cm^−2^ were used in all cases. Each experiment was repeated three times with pristine CCMs and transport layers, and the mean value including standard deviation is plotted (note that in some instances the data point overlays the error bar). Electrode loadings are given as mean values including min/max deviation.

In **Figure** **7**a,b, the polarization curves of CCMs with the core–shell and the Umicore catalyst are displayed together with the high‐frequency resistance (HFR) and HFR‐corrected polarization curves. Figure 7a shows the baseline experiment with a relatively high iridium loading of ≈1.3 mg_Ir_ cm^−2^. As expected from the in‐plane conductivity measurements (Figure S14, Supporting Information), the core–shell CLs exhibit a higher ohmic resistance (as seen from the steeper slope of the polarization curve) and a higher HFR than the Umicore CL.

For the HFR‐free polarization curves, the performance of the two CCMs is similar. This leads us to the conclusion that the performance difference mainly stems from resistances included in the HFR. Since all components, except the anode catalyst layer, are kept the same, contributions to the resistance stem from the anode CL bulk and interfacial contacts between the anode CL and PTL. An additional bulk electrical resistance of the core–shell CL (as opposed to the Umicore CL) could stem from the lower iridium content^[^ ^50^ ^]^ and decreased conductivity of amorphous IrO_x_.^[^ ^51^ ^]^ Additionally, two structural factors have an impact. First, the electrical pathway is longer as the CL thickness for the core–shell catalyst is significantly increased compared to the Umicore catalyst for the same absolute iridium loading (core–shell ≈14 µm vs Umicore ≈5 µm at 1.3 mg cm^−2^_;_ from thickness factor in Figure 6a). Second, the cross‐sectional area for electrical conduction is reduced since it only takes place via the thin shell for the core–shell CL. This combination leads to the inferior overall performance of the core–shell CL compared to the CCM with Umicore catalyst at high loadings (filled symbols in Figure 7a).

However, the HFR‐corrected performance at low current densities of the core–shell catalyst is superior to the reference (see inset Figure 7a). This can be assigned to a higher intrinsic activity of the substoichiometric and amorphous IrO_x_ of the core–shell catalyst compared to the rutile IrO_2_ of the Umicore catalyst, as evident from RDE measurements (Figure 4a). In contrast, starting from 3 A cm^−2^, the HFR‐free polarization curves are crossing. This could be explicable by two scenarios.

First, mass transport issues could lead to an upward bending of the polarization curve due to additional voltage losses. A difference in pore size is apparent from the FIB‐SEM cross sections in Figure 5d,e. Besides the pore size, the wettability and surface structure inside the pores of the material were shown to influence the bubble formation and the resulting two‐phase flow of water and oxygen.^[^ ^52^, ^53^ ^]^ Further investigation is required to determine the influence of these parameters and their interaction. A second explanation could be an increased ionic resistance of the catalyst layer (not included in the HFR^[^ ^54^ ^]^), resulting in a larger slope of the HFR‐free polarization curve for the core–shell CL. When keeping the absolute iridium loading constant, the CL thickness for the core–shell catalyst is significantly increased compared to the Umicore catalyst (≈14 µm vs ≈5 µm at 1.3 mg cm^−2^). Even though the tortuosity of the ionic pathway within the core‐shell CL seems to be decreased (compare Figure 5d and 5e), the threefold increased thickness could lead to a longer ionic percolation path resulting in a higher resistance (as sketched in blue in Figure S15, Supporting Information).

When decreasing the loading to 0.2–0.3 mg_Ir_ cm^−2^, as shown in Figure 7b, the core–shell catalyst noticeably outperforms the Umicore reference catalyst throughout the full current–density range. The CCMs with the core–shell catalyst show a decreased cell potential by 50 mV @ 2 A cm^−2^ compared to the reference CCMs (1.77 V vs 1.82 V @ 2 A cm^−2^). Regarding the HFR values, opposite trends can be observed. While the CCMs with the Umicore catalyst on the anode CL exhibit an increased HFR for low loadings (as already observed in the literature^[^ ^8^ ^]^), the HFR of the CCMs with the core–shell catalyst decreases with decreasing loading.

For the low‐loading case, the CLs are thinner (core–shell ≈2.4 µm vs Umicore ≈1.1 µm), which influences the performance of the two CLs in different ways. In the case of the Umicore catalyst, we assign the observed decrease in performance (Figure 7b) to the cracking of the layer upon membrane swelling during operation. Suppose the layer thickness decreases below the threshold of 2 µm. In that case, cracks can lead to a disconnection of the CL and a decreased in‐plane conductivity, resulting in a higher electron transport resistance within the CL. In combination with a relatively coarse PTL with large pores that do not compensate for a disconnection, this can explain an increased HFR with decreased loading.^[^ ^8^ ^]^

When using uncoated titanium PTLs, the native TiO_2_ layer acts as a semiconductor and might induce a Schottky barrier between CL and PTL, thereby adding resistance.^[^ ^55^ ^]^ However, such a band bending is probably not applicable to explain an increased HFR in our case because platinum‐coated PTLs are used in all measurements. With Pt coating (metal–metal contact between PTL and CL), no Schottky barrier should be present at the PTL|CL interface, resulting in the same effect for both catalysts independent of their oxidation state and work function. This was also observed for a system with rutile IrO_2_ or amorphous IrO_x_, where the implementation of a platinum coating on the PTL resulted in a very similar HFR of 50 and 47 mΩ cm^2^ for the rutile and amorphous iridium oxide catalyst, respectively.^[^ ^13^ ^]^

It could be argued that the advantage of decreasing the iridium loading is diminished when the PTL needs to be coated with Pt, as additional platinum group metal (PGM)‐content is added to the system. However, in our work, the mean amount of added Pt to each cell yields 0.035 ± 0.025 mg_Pt_ cm^−2^, while the tenfold decrease in iridium loading compared to a state‐of‐the‐art loading of 2 mg cm^−2^ saves 1.8 mg_Ir_ cm^−2^. Additionally, platinum is more abundant than iridium, so it is currently not the bottleneck for a scale‐up of PEMWE.^[^ ^4^ ^]^

Comparing the high‐ and low‐loading performance of the core–shell CL, the kinetic penalty due to a decreased loading is balanced out by a decrease in HFR, leading to superior performance compared to the reference CL. For current densities higher than 2 A cm^−2^, the low‐loading CL even outperforms the high‐loading CL with the core–shell catalyst (1.99 vs 2.02 V at 4 A cm^−2^). The decrease in HFR for the low‐loading core–shell CL is assigned to a combination of two phenomena: First, layer cracking is prevented due to a higher thickness factor. Second, the electrical resistance of the CL is reduced from high‐ to low‐loaded CLs due to the thickness reduction and, as a result, a shorter electrical pathway. In this regard, an optimal layer thickness needs to be determined.

A comparison of the HFR‐free polarization curves at low iridium loadings shows the higher activity of the core–shell catalyst, outperforming the CCM with the Umicore catalyst. The HFR‐free polarization curves maintain a constant difference. Thus, neither the ionic resistance contribution nor a mass transport limitation appears in the low‐loading case with the core–shell catalyst, where the layer thickness is reduced compared to the high‐loading case (≈14 vs ≈2.4 µm).

The HFR‐free performance of the Umicore catalyst with low loading is decreased compared to the high‐loading case. This could stem from the cracking of the CL, leading to parts of the CL not being active and thus increasing the kinetic overpotential.^[^ ^56^ ^]^ Note that the deviations in the case of low loading with IrO_2_/TiO_2_ Umicore CL are larger (Figure 7b, grey curves), hinting toward an issue with the homogeneity of the electrodes in general, which is not observed for higher loadings or any loading with our TiO_2_@IrO_x_ core–shell catalyst.

Moreover, Figure 7a,b displays an increase in HFR with current density for the core–shell CL for both, high and low loadings. In general, this can be caused by either in‐plane or through‐plane effects. In‐plane inhomogeneities can cause an apparent higher bulk electrolyte resistance either by local dry out of the membrane (which can be caused by large land structures of the PTL^[^ ^57^ ^]^) or by inhomogeneous usage of the bulk electrolyte phase for the ion transport. The latter is often observed for alkaline water electrolysis systems where bubble accumulation can block direct ionic pathways between the electrodes.^[^ ^58^ ^]^ However, also for membrane‐based electrolysis systems, such an effect can be envisioned if the evolved gases accumulate in larger domains of the CL (i.e., on the same scale as the bulk electrolyte thickness). Such domains could then not contribute to the overall reaction rate efficiently, leading to inhomogeneous catalyst utilization and therefore, a higher HFR. Minding the larger pore size for the core–shell CL, however, we deem this effect not to be the cause for the observed trend, especially in comparison to the nano‐porous reference CL.

On the other hand, the through‐plane effects of the membrane could be the reason for the behavior of the core–shell CL HFR. The high osmotic drag at high current densities could lead to reduced humidification close to the interface of the anode CL with the membrane. While a change in water profile across the membrane with current density was shown for thick membranes,^[^ ^59^ ^]^ the same effect can also be explained for thin membranes (as in our case ≈51 µm) paired with thick electrodes.^[^ ^8^ ^]^ At high current densities, the void volume of the anode CL will be filled with the oxygen product gas, limiting the water transport toward the active site to the ionomer film in the CL. Due to the larger thickness of the core–shell CL, this increase in HFR with higher current densities is apparent in Figure 7a,b (green curves).

The stability of the newly developed core–shell catalyst and the commercial references (IrO_x_ Alfa Aesar Premion and IrO_2_/TiO_2_ Umicore Elyst Ir75) was examined in a scanning flow cell (SFC) coupled to an inductively coupled plasma mass spectrometer (ICP‑MS) as well as in single‐cell testing. In the SFC‐ICP‐MS measurement (Figure S16, Supporting Information), the commercial catalyst from Umicore exhibits the highest dissolution stability, which is attributed to the complete oxidation to rutile IrO_2._ Promisingly, the stability of the TiO_2_@IrO_x_ core–shell catalyst is similar to the Alfa Aesar reference catalyst. The same trend between the core–shell catalyst and the reference from Umicore is observed from the single‐cell stability testing over 200 h @ 2 A cm^−2^ (Figure S17, Supporting Information). We observe a higher degradation rate for the core–shell catalyst compared to the reference from Umicore. As analyzed in Figure S18a (Supporting Information), the main contribution to the degradation rate stems from an increase in HFR‐free potential. STEM‐EDXS measurements before and after the 200  h current hold (Figure S18b,c, Supporting Information) reveal a decrease in the iridium‐to‐oxygen ratio throughout the current hold. These two results hint toward a further oxidation of the catalyst material throughout the current hold. Therefore, we hypothesize a decrease in degradation rate with prolonged testing time.

Compared to various literature values for low iridium loading PEMWE stability testing with newly developed catalysts, the degradation rate of our catalyst is in a similar range (cf. Section S2.2 Stability testing, Supporting Information).^[^ ^10^, ^21^ ^]^ Additionally, the core–shell structure was examined via HAADF‐STEM after 200 h of testing. As shown in Figure S19 (Supporting Information), the particles appear unchanged. It can be concluded that the catalyst already possesses good stability, which needs to be further improved.

To directly compare the technological suitability of the two catalysts, the iridium‐specific power density can be used. It is calculated based on the current density reached at a cell voltage of 1.79 V (equals 70% efficiency with regard to the lower heating value).^[^ ^1^, ^8^ ^]^ With the values from Figure 7b (filled symbols, not HFR‐corrected), the core–shell catalyst possesses an iridium‐specific power density of 17.9 kW g_Ir_^−1^, which is 1.7‐times higher than the Umicore catalyst with a power density of 10.4 kW g_Ir_^−1^. However, the value is still ≈3–6 times lower than the postulated mass activity of 50–100 kW g_Ir_^−1^ needed for a successful large‐scale implementation of PEMWE.^[^ ^7^, ^8^, ^9^ ^]^ Nevertheless, there is potential to further decrease the loading of the core–shell catalyst without disconnecting the catalyst layer by reducing the iridium content in the catalyst powder below 40 wt.%, which is currently under investigation.

Decreasing the iridium loading in the anode catalyst layer for single‐cell measurements was the subject of several previous studies where various approaches were tested. Since it is difficult to directly compare results across labs due to differences in cell hardware, components, and operation conditions, the examples here should serve as a context in the effort to employ low iridium loadings. The examples are further used in the comparison for our stability test results (cf. Section S2.2 Stability testing, Supporting Information). In an earlier publication from our group, TiO_2_ microparticles were coated with IrO_2_ and used to prepare porous transport electrodes. At a loading of 0.4 mg_Ir_ cm^−2^ the developed catalyst outperformed the reference from Umicore IrO_2_/TiO_2_ with 0.5 mg_Ir_ cm^−2^.^[^ ^21^ ^]^ Hegge et al.^[^ ^10^ ^]^ implemented an IrO_x_‐nanofiber interlayer between an IrO_x_‐nanoparticle‐based CL and the PTL to improve their interfacial electrical contact and combine high porosity (interlayer) and high surface area (nanoparticles) within the CL. With an overall low loading of 0.2 mg_Ir_ cm^−2^, it was possible to achieve a similar performance compared to a cell with 1.2 mg_Ir_ cm^−2^ of only IrO_x_ nanoparticles.^[^ ^10^ ^]^ Taie et al.^[^ ^49^ ^]^ were able to decrease the loading of a bulk IrO_2_ catalyst down to 0.026 mg_Ir_ cm^−2^ while observing a better performance than a 2.5 mg_Ir_ cm^−2^ loaded commercial reference MEA (Greenerity E400). They argue that controlling the CL morphology makes low loadings possible, which they achieve by preparing CCMs via direct CL deposition onto the membrane with spray coating. Möckl et al.^[^ ^5^ ^]^ tested a hydrous iridium oxide supported on TiO_2_ with a loading of 0.25 mg_Ir_ cm^−1^. The new catalyst clearly outperformed the Umicore IrO2/TiO_2_ benchmark catalyst at a state‐of‐the‐art loading of 2 mg_Ir_ cm^−2^. A summary of Ir‐specific power density and durability of catalysts in recent publications can be found in Table S4 (Supporting Information).

These few examples show that there are several different approaches in research to decrease the anode catalyst loading for a scale‐up of the PEMWE technology. Our performance is not in all cases superior to the presented literature examples. However, we emphasize that our synthesis route is facile and scalable, and the coating method used to prepare electrodes is industrially applicable. Besides, our approach is open to being combined with other approaches, for example, the implementation of an interlayer (cf. Hegge et al.^[^ ^10^ ^]^) to improve interfacial contact and cope for the decreased (in‐plane) conductivity of our core–shell catalyst.

## 3 Conclusion

In this work, we successfully developed a photodeposition synthesis method to obtain TiO_2_@IrO_x_ core–shell particles with an iridium content as low as 40 wt.%. These core–shell particles are unique because of their thin (2.1 ± 0.4 nm) and homogeneous shell of iridium oxide. The formation mechanism of this thin shell was analyzed in detail and is attributed to the combination of seed formation during photodeposition and closing of the shell during annealing.

In electrochemical three‐electrode testing, the TiO_2_@IrO_x_ catalyst exhibits both sufficiently high mass activity and decent stability compared to commercial catalysts. Notably, the powder conductivity of the core–shell catalyst was increased threefold with the shell compared to the catalyst without the shell. This increase is attributed to an improved electrical percolation because of the continuous shell. This finding combined with the novel synthesis method paves the way toward iridium core–shell catalysts with sufficient conductivity at low iridium contents.

Due to the low iridium content of 40 wt.% in the catalyst powder, a high catalyst layer thickness factor of 10.9 ± 1.1 µm (mg cm^−2^)^−1^ is achieved, which allows the fabrication of sufficiently thick catalyst layers even at low loadings of 0.22 mg_Ir_ cm^−2^.

The resulting low‐loaded CCM outperforms the commercial catalyst in terms of both, activation overpotential and HFR. A possible explanation is that a thicker catalyst layer, in the case of the core–shell CCM, prevents CL cracking during operation and thus maintains the in situ in‐plane conductivity. The reference CCM, on the contrary, exhibits a thin catalyst layer, which is prone to cracking and which could explain the decreased performance.

Altogether, the combined improvements of the TiO_2_@IrO_x_ core–shell CL lead to a high single‐cell performance of 1.77 V and 1.98 V at 2 and 4 A cm^−2^, respectively, at a low iridium loading of 0.22 mg_Ir_ cm^−2^. Importantly, the core–shell structure of the catalyst was maintained after 200 h of single‐cell operation. This novel TiO_2_@IrO_x_ core–shell catalyst exhibits an iridium‐specific power density of 17.9 kW g_Ir_^−1^, which is almost twice as high as the reference catalyst from Umicore, bringing PEMWE technology closer to the goal of 50–100 kW g_Ir_^−1^ while at the same time using well‐known material systems and scalable processes.

## 4 Experimental Section

### Synthesis of TiO_2_@IrO_x_ Core–Shell Particles

The synthesis of the TiO_2_@IrO_x_ particles with 40 wt.% iridium is based on three process steps which are elucidated below. For the reaction solution, 1.39 g of IrCl_3_ ∙ H_2_O (53.7% iridium, 99.8% purity (metals basis), Thermo Fisher Scientific) were dissolved in 50 mL deionized (DI) water (18.2 MΩ cm) and the pH value was adjusted to 11 with 1 m KOH. In a second flask, 1.12 g of TiO_2_ (rutile, < 5 µm, > 99.9% (trace metals basis), Sigma–Aldrich) were dispersed in 100 mL DI water and 1 mL of 1 m KOH (pH 11) by ultrasonication in an ultrasonic bath for 20 min. Both solutions were mixed, and 30 mL of isopropanol (> 99.8%, Sigma–Aldrich) was added as a hole scavenger.

The first reaction step was the photodeposition, for which the reaction solution was exposed to UV–C radiation (254 nm wavelength) from a laboratory lamp (UVP Multiple Ray Lamp, 8 W, Analytik Jena) with a power density of 10–12 mW cm^−2^ at the solution surface (measured with UVpro Radiometer, Nuvonic GmbH) for 24 h. Second, the UV radiation was switched off, and the reaction solution was heated at 100 °C under reflux for 1 h. After cooling down to room temperature, the solution was filtered, yielding a clear filtrate and a black powder, indicating that all the iridium precursor was consumed. During filtering, the powder was washed with 1 L of DI water. After drying the powder at 70 °C for at least 4 h and grinding it with an agate mortar for 10 min, the catalyst powder underwent an annealing step as the third part of the synthesis. The powder was heated to 350 °C at 10 K min^−1^ and held at the target temperature for 30 min under a continuous flow of synthetic air (100 mL min^−1^).

### Physical Characterization of the Catalyst Powder

The synthesized TiO_2_@IrO_x_ catalyst material was studied thoroughly using different physical characterization methods such as transmission electron microscopy (TEM) (including high‐resolution TEM (HRTEM), high‐angle annular dark field scanning TEM (HAADF‐STEM)), energy dispersive X‐ray spectroscopy (EDXS) and electron diffraction (ED), X‐ray photoelectron spectroscopy (XPS), X‐ray diffraction (XRD), X‐ray fluorescence (XRF), powder conductivity measurements and N_2_‐physisorption analysis. From the N_2_‐physisorption analysis, the specific surface area was determined using the Brunauer–Emmett–Teller (BET) method.^[^ ^60^ ^]^ The experimental details of those measurements are given in the Supporting Information. As a reference, the powder conductivity and the surface area (via N_2_‐physisorption) were also determined for the two commercial catalysts, IrO_x_ (Alfa Aesar Premion) and IrO_2_/TiO_2_ (Umicore Elyst Ir75 0480).

### Electrochemical Characterization of the Catalyst Powder

The electrochemical performances of the synthesized TiO_2_@IrO_x_ catalyst, the synthesized catalyst without shell (denoted as IrO_x_/TiO_2_), and the two commercial references IrO_x_ (Alfa Aesar Premion), and IrO_2_/TiO_2_ (Umicore Elyst Ir75 0480) were characterized in rotating disc electrode (RDE) measurements. Additionally, the iridium dissolution during electrochemical testing was measured with a scanning flow cell (SFC) coupled on‑line to an inductively coupled plasma mass spectrometer (ICP‑MS). Both measurement techniques were performed in Argon‐purged 0.1 m HClO_4_ at room temperature and a loading of 50 and 10 µg_Ir_ cm^−2^ for RDE and SFC‐ICP‐MS measurements, respectively. The experimental details of both techniques are given in the Supporting Information.

### Catalyst Layer Fabrication and their Structural Analysis

To evaluate the performance of the new catalyst in a single cell, catalyst‐coated membranes (CCMs) were prepared using a standard decal method. The synthesized TiO_2_@IrO_x_ catalyst material was compared to the commercial IrO_2_/TiO_2_ (Umicore Elyst Ir75 0480) catalyst. All CCMs consisted of an anode with either the core–shell or the Umicore catalyst, a Nafion NR212 membrane, and a Pt/C cathode. Details on the CCM fabrication can be found in the Supporting Information.

The structure of the TiO_2_@IrO_x_ catalyst layers was further analyzed by TEM on a lamella prepared via a focused ion beam (FIB) and by nano X‐ray computed tomography (nano‐CT). A structural comparison of the catalyst layers with the core–shell TiO_2_@IrO_x_ catalyst and the commercial IrO_2_/TiO_2_ catalyst was done by FIB scanning electron microscopy (SEM) and cross‐sectional imaging. The electrical conductivity of catalyst layers prepared with those two different catalysts was measured additionally. Details on the structural analysis of the layers can be found in the Supporting Information.

### Single‐Cell Tests in PEMWE Setup

All cell tests with CCMs were performed on a commercial test bench (600 Electrolyzer Test System, Scribner LLC) equipped with a potentiostat with a current booster (BioLogic VSP‐300). An in‐house designed cell fixture adapted from previous reports^[^ ^48^ ^]^ with a 5 cm^2^ active area was used. Two sets of CCMs were manufactured for both catalysts. One set with high‐loading anodes of 1.33 ± 0.20 mg_Ir_ cm^−2^ with the core–shell TiO_2_@IrO_x_ and the reference IrO_2_/TiO_2_ catalyst (Umicore Elyst Ir75), respectively. For the low‐loading set, anodes with 0.22 ± 0.01 and 0.30 ± 0.03 mg_Ir_ cm^−2^ for core–shell and Umicore catalyst were fabricated, respectively. For all CCMs, the cathode loading was kept to 0.19 ± 0.08 mg_Pt_ cm^−2^.

The respective CCM was assembled with a platinum‐coated porous transport layer (PTL) consisting of titanium (2GDL10‐0,25 from NV Bekaert SA, Belgium) on the anode and a gas diffusion layer (GDL) with a microporous layer (MPL) consisting of carbon (H24C5 Freudenberg & Co. KG) on the cathode. The PTL was platinum coated on both sides via sputter deposition (Cressington sputter coater 108) with an average loading of 0.035 ± 0.025 mg_Pt_ cm^−2^. Gasket thicknesses were chosen so that a compression of the carbon GDL of 23.7 ± 2.2% was achieved. The anode was flushed with 100 mL min^−1^ DI water which was additionally purged with nitrogen for 10 min prior to testing to remove any dissolved CO_2_ from refilling of the tank. The anode feed was heated to 80 °C, the cathode was kept dry and only the venting line behind the cell was purged with nitrogen for safety reasons. The cell was heated to 80 °C with cartridge heaters placed into both endplates and regulated by a thermocouple placed into the cathodic flow field plate. Temperatures were kept constant throughout the whole testing time.

When the cell temperature and anode feed reached 80 °C, the temperature was held for 30 min before starting the electrochemical testing. The test begins with a test for electrical short at 1 V for 1 min and a conditioning step of 30 min at 1 A cm^−2^. In the following, three subsequent polarization curves were recorded. The polarization curves are measured between 0.01 and 4 A cm^−2^, holding each current density step for 5 min to ensure steady‐state conditions and averaging the last 15 s for analysis. Galvanostatic electrochemical impedance spectroscopy (EIS) was performed from 100 kHz to 500 mHz at each point of the polarization curve after the respective current density hold. The perturbation of each AC current was chosen ≤10% of the AC current but not smaller than 20 mA so that a good signal‐to‐noise ratio was achieved and a linear system response was ensured. Since the first two polarization curves were considered part of conditioning, an overlap of the last two curves was checked, and only the last polarization curve was taken for analysis. All tests were repeated three times with pristine CCMs for reproducibility.

### Data Evaluation from Cell Tests

High‐frequency resistance (HFR) values were extracted from the electrochemical impedance measurements at each current density by fitting a frequency interval of 50–0.5 kHz with an equivalent circuit model comprising an inductance, a resistor, and a transmission line model in series.^[^ ^54^, ^61^ ^]^ Adjusted R^2^ (R^2^_adj_ ≥ 0.99) was used as a quality indicator for the fit. Fitting was performed using an in‐house developed Python routine based on NumPy,^[^ ^62^ ^]^ SciPy,^[^ ^63^ ^]^ pandas,^[^ ^64^, ^65^ ^]^ matplotlib,^[^ ^66^ ^]^ and impedance.py.^[^ ^67^ ^]^ An exemplary Nyquist plot with fit can be found in Figure S1 (Supporting Information). HFR‐free polarization curves were derived by subtracting the product of HFR and current density from the polarization curve. A Tafel analysis was not conducted in the single‐cell measurements since, for the tests with low loadings, the Tafel requirements were not fulfilled in the lowest current density region and measuring to even lower current densities was not feasible due to resolution limitations.

## Conflict of Interest

A patent application has been filed based on parts of this study. No further conflict of interest is to be declared.

## Author Contributions

D.H. and S.F. contributed equally to this work. D.H. conceptualized the study, performed formal analysis, and investigation, developed methodology, validated the study, performed visualization, wrote the original draft, and wrote, reviewed, and edited the final draft. S.F. conceptualized the study, performed formal analysis, and investigation, developed methodology, validated the study, performed visualization, wrote the original draft, and wrote, reviewed, and edited the final draft. L.F. performed the investigation, and formal analysis, wrote, reviewed, and edited the final draft. T.‐C.M. performed investigation, and wrote, reviewed, and edited the final draft. A.K. performed the investigation, and wrote, reviewed, and edited the final draft. M.Z. performed the investigation, and formal analysis, wrote, reviewed, and edited the final draft. K.W.‐B. performed the investigation, and formal analysis, wrote, reviewed, and edited the final draft. S.C. performed the investigation, and formal analysis, wrote, reviewed, and edited the final draft. A.G. performed formal analysis, and wrote, reviewed, and edited the final draft. B.A.Z. performed formal analysis, acquired funds, supervised the study, and wrote, reviewed, and edited the final draft. J.W. performed formal analysis and supervision. E.S. acquired funds, supervised the study, and wrote, reviewed, and edited the final draft. B.F. performed investigation, and formal analysis, developed methodology, worked with software, and wrote, reviewed, and edited the final draft. S.C. developed the methodology, supervised the study, and wrote, reviewed, and edited the final draft. A.T.S.F. conceptualized the study, developed the methodology, and wrote, reviewed, and edited the final draft. K.J.J.M. conceptualized the study, acquired funds and resources, supervised the study, and wrote, reviewed, and edited the final draft. S.T. conceptualized the study, acquired funds, performed project administration, acquired resources, supervised the study, and wrote, reviewed, and edited the final draft. A.H. conceptualized the study, acquired funds, performed project administration, formal analysis, investigation, and supervision, and wrote, reviewed, and edited the final draft. C.v.P. conceptualized the study, acquired funds, supervised the study, and wrote, reviewed, and edited the final draft.

Supporting Information includes detailed experimental procedures (catalyst‐coated membrane fabrication, transmission electron microscopy, X‐ray photoelectron spectroscopy, X‐ray diffraction, powder conductivity, N2‐physisorption, nuclear magnetic resonance, nano X‐ray computed tomography, focused ion beam scanning electron microscopy, SEM cross‐sectional imaging, electronic in‐plane conductivity, rotating disk electrode, scanning flow cell coupled to an inductively coupled plasma mass spectrometer), tips for reproducing the photodeposition synthesis, stability testing of the TiO2@IrOx catalyst, Figures S1–S19 and Tables S1 and S4.

## Supporting information

Supporting Information

Supplemental Video 1

Supplemental Video 2

Supplemental Video 3

Supplemental Video 4

## Data Availability

The data that support the findings of this study are openly available in Zenodo at doi.org/10.5281/zenodo.10658498, reference number 10658498.
