# Supplementary material for: Photodeposition‐Based Synthesis of TiO2@IrOx Core–Shell Catalyst for Proton Exchange Membrane Water Electrolysis with Low Iridium Loading
Source: Adv Sci (Weinh). 2024 Jun 14;11(30):2402991. doi: 10.1002/advs.202402991 (PMC11321668; doi:10.1002/advs.202402991)
Supplement: Supplementary file 1 — Supporting Information [file ADVS-11-2402991-s005.docx]

Supporting Information

Photodeposition-based Synthesis of TiO_2_@IrO_x_ Core-Shell Catalyst for Proton Exchange Membrane Water Electrolysis with Low Iridium Loading

Darius Hoffmeister, ^‡,^* Selina Finger, ^‡,^* Lena Fiedler, Tien-Ching Ma, Andreas Körner, Matej Zlatar, Birk Fritsch, Kerstin Witte‑Bodnar, Simon Carl, Alexander Götz, Benjamin Apeleo Zubiri, Johannes Will, Erdmann Spiecker, Serhiy Cherevko, Anna T. S. Freiberg, Karl J. J. Mayrhofer, Simon Thiele, Andreas Hutzler* and Chuyen van Pham*

^‡^ These authors contributed equally to this work.

**1. Experimental Section**

*Catalyst-coated membrane (CCM) fabrication:* Catalyst-coated membranes (CCMs) were manufactured *via* the decal transfer technique where 5 cm^2^-sized electrodes coated onto a PTFE substrate are united with a ~ 51 µm thick Nafion^TM^ NR212 membrane (Chemours) by a hot-pressing step at 155 °C and 2.5 MPa. The loadings of the electrodes were determined by weighing the decals before and after hot pressing.

The catalyst inks were prepared from a mixture of the respective catalyst powder with suitable solvents and Nafion^TM^ D2021 ionomer dispersion (Chemours). For the anode, the in-house synthesized TiO_2_@IrO_x_ catalyst powder was mixed with DI water (18.2 MΩ cm), isopropanol (> 99.8%, Sigma-Aldrich), and the ionomer dispersion with a water to solvent ratio of 6.19 wt%, an ionomer to catalyst ratio of 12.99 wt% and a total solid content of 0.46 g ml_ink_^-1^.

For the reference experiments, a commercially available IrO_2_/TiO_2_ catalyst (Elyst Ir75 0480, Umicore) was used on the anode side and mixed with 1‑propanol (> 99.5%, Sigma-Aldrich) and the ionomer dispersion with an ionomer to catalyst ratio of 13.12 wt% and a total solid content of 0.46 g ml_ink_^-1^. The average ionomer film thickness in wet conditions is estimated to be ~ 80 nm and ~ 2 nm for the core-shell and Umicore case, respectively.

For the cathode in both cases, platinum supported on carbon catalyst powder (Pt/C, TEC10V40E from Tanaka, Japan) was mixed with DI water, 1‑propanol and the ionomer dispersion. The inks were suspended for 24 h using a roller mixer (BTR5-12V, Ratek) with zirconium beads (5 mm diameter, Fritsch^TM^ GmbH) at 180 rpm and 60 rpm for anode and cathode inks, respectively. From the inks, electrodes were coated onto 50 µm thick PTFE substrate (High-tech-flon) using the Mayer rod technique (rods from ERICHSEN GmbH & Co. KG). A variation of loadings was achieved by using rods with different wire sizes. The electrode sheets were dried for 2 h at 70 °C (BINDER oven) before being cut into 5 cm^2^ electrodes and hot pressed with the membrane.

- 1. **Physical characterization**

*Transmission electron microscopy (TEM):* To gain insight into the nanostructure of the catalysts, TEM samples were prepared by depositing small amounts of the catalyst on a TEM grid (Lacey carbon on Ni grid, TedPella). For the imaging of the end-of-test catalyst particles (cf. **Figure S19**), parts of the anode catalyst layer were scratched off with a scalpel, redispersed in a mixture of water and isopropanol and deposited on a TEM grid. Plasma cleaning was performed for samples without Nafion using a PIE Scientific Tergeo-EM Plasma Cleaner utilizing ambient air remote plasma, whereas end-of-test samples were not plasma cleaned.

A Talos F200i from Thermo Fisher Scientific equipped with a Schottky emitter (X-FEG) and a Dual Bruker XFlash 6T-100 EDS detector was used for high-angle annular dark field scanning transmission electron microscopy (HAADF-STEM) and spectrum imaging employing energy dispersive X-ray spectroscopy (EDXS) at a primary electron energy of 200 keV. Micrographs have been processed using Velox (v. 3.9.0) and FIJI (FIJI is just ImageJ).^[1]^

For further TEM analysis, a thin TEM lamella of a catalyst layer with 0.2 mg_Ir_ cm^-2^ loading (cf. **Figure 3**a-c) was prepared using a Helios Nanolab 660 dual beam focused ion beam scanning electron microscope (FIB-SEM) from Thermo Fisher Scientific. The Ga^+^ milling was performed in three subsequent milling steps at 30 kV, 8 kV, and 2 kV acceleration voltage with 0.79 nA, 0.12 nA, and 23 pA ion beam current, respectively. TEM analysis of this FIB cross section was performed on a Titan³ Themis 60-300 double aberration-corrected TEM (Thermo Fisher Scientific, USA, equipped with C_s_-correctors both at the probe and imaging side) with an acceleration voltage of 300 kV in high-resolution transmission electron microscopy (HRTEM) mode with 190 pA last measured screen current. Electron diffraction (ED) patterns were acquired using 580 mm camera length and 1.75 nA last measured screen current. HRTEM micrographs have been processed using Velox (v. 3.9.0). ED pattern identification was performed in JEMS (Java electron microscopy software, Version 4.8330U2019b20) and the crystallographic data from the Inorganic Crystal Structure Database (ICSD) with collection code #53183 was used.

*Nuclear magnetic resonance (NMR):* ^1^H NMR spectra of the reaction solution after the photodeposition step, the reaction solution after both steps, photodeposition and IPA reduction, and a reference sample of an H_2_O/IPA mixture were collected. A 500 MHz JEOL JNM-ECZR spectrometer equipped with a ROYALPROBE HFX with decoupling was used for this.

*X-ray fluorescence (XRF):* The iridium content of the synthesized catalyst was determined using a Bruker M4 Tornado XRF with a rhodium source at a voltage of 50 kV. The ratio of iridium to TiO_2_ was measured at ten different spots and averaged to give the iridium content of the catalyst.

*X-ray photoelectron spectroscopy (XPS):* The iridium surface oxidation state was analyzed using a Thermo Fisher Nexsa G2 XPS at a pressure of ~ 10^‑8^ mbar and an aluminum anode with a computer-controlled quartz crystal monochromator leading to an excitation energy of 1486.6 eV (Al Kα line). A survey spectrum was taken to compensate for influences from sample charging, and the spectrum was calibrated so that C1s peak was centered at 284.8 eV.^[2]^ Afterwards, a detailed spectrum of the Ir 4f double peak was acquired with a step size of 0.1 eV and 50 ms dwell time per energy set point.

The fitting of the Ir 4f spectra was performed with the software CasaXPS.^[3]^ First, a Shirley background was subtracted and then Ir(0), Ir(IV), and Ir(III) peaks were fitted using a doublet splitting of 3 eV and a typical area ratio of 4:3 between the 4f_7/2_ and the 4f_5/2_ peaks. The binding energies of the different peaks, the line shapes, and the typical full-width at half maximum (FWHM) values were chosen according to the literature.^[4–6]^ The detailed fitting parameters are shown in Table S1.

*X-ray diffraction (XRD):* The phase and crystallite size of the iridium catalyst were investigated by conducting XRD measurements on a Bruker D8 Advance equipped with a Lynxeye XE-T detector. A Cu Kα source (8047.8 eV) was used and the resulting diffractograms were analyzed using the Rietveld refinement method implemented in the software Profex.^[7]^ PDF #04-003-0648 and PDF #04-007-0701 from the International Centre for Diffraction Data (ICDD) database were used for TiO_2_ rutile and TiO_2_ anatase, respectively. For iridium, the crystallographic data #9008470 from the Crystallography Open Database (COD) was used.^[8]^

*Powder conductivity:* Powder conductivity measurements were conducted in a 3D-printed setup as described in detail in Fraser *et al*.^[9]^ In brief, 20 mg of catalyst powder was compressed with a pressure of 3 MPa by a tensile tester in compression mode (Shimadzu EZ-SX). This compression resulted in catalyst pallet thicknesses (only dry powder) between 150 and 290 µm depending on the catalyst. Four electrodes embedded in the punch were used to contact the sample and a voltage $V_{12}$ was applied between electrode 1 and 2. The resulting current $I_{12}$ was measured while simultaneously measuring the voltage drop $V_{34}$ between electrodes 3 and 4. For all electrical measurements, the built-in source measure unit of a four-point probe (Ossila B. V., Four-Point Probe T2001A3) was used. The electrical resistance *R*_12,34_ was calculated using Ohm’s law:

| $R_{12,34}=\frac{V_{34}}{I_{12}}$ | (1) |
| --- | --- |

The thickness of the compressed sample *d* was accurately measured by the tensile tester. Thus, the van-der-Pauw method of calculating the resistivity $\rho$ was employed^[10]^:

| $\rho=\frac{\pi d}{\ln2}\cdot\frac{R_{12,34}+R_{23,41}}{2}\cdot f$ | (2) |
| --- | --- |

where $R_{23,41}$ indicates a cyclic rotation of the electrode contacting and *f* is a geometric correction factor to account for a difference in $R_{12,34}$ and $R_{23,41}$. It is noted that Eq. 2 is an analytical approximation of the van-der-Pauw equation under the assumption that the material exhibits an isotropic conductivity,^[11]^ which is expected for a powder sample with close to spherical particles.

During testing, it was found that in this work:

| $R_{12,34}\approx2\cdot R_{23,41}$ | (3) |
| --- | --- |

which is attributed to a non-equidistant electrode spacing due to fabrication tolerances. This factor of 2 results in a geometric correction factor of $f\approx1$.^[11]^ Taking these findings into account, Eq. 2 can be simplified to

| $\rho=3.399\cdot d\cdot R_{12,34}$ | (4) |
| --- | --- |

For every catalyst, three samples were measured for statistical relevance and the powder conductivity was reported as

| $\sigma=\frac{1}{\rho} .$ | (5) |
| --- | --- |

*N_2_-physisorption:* The specific surface area of the powder samples was determined *via* N_2_-physisorption measurements at 77 K (Micromeritics TriStar II Plus) and evaluated using the Brunauer-Emmett-Teller (BET) method^[12]^ at partial pressures between 0.1 and 0.5 p/p_0_. Before measuring, samples were degassed for 24 h at 423 K and < 0.05 mbar.

*Nano X-ray computed tomography (nano-CT):* The catalyst volume fraction and the pore-size distribution (pores include void volume and ionomer film due to method) of the anode catalyst layer with the core-shell catalyst were determined *via* nano X-ray computed tomography (nano-CT). The term porosity is avoided here since this method cannot distinguish ionomer and void volume. The measurement was performed with a ZEISS Xradia 810 Ultra X-ray microscope equipped with a 5.4 keV rotating Cr anode source and a Zernike phase ring for phase contrast (PC) imaging.

A cutout (~ 7 mm ∙ 15 mm) of a pristine CCM (with anode loading of 0.2 mg_Ir_ cm^-2^) was prepared with a scalpel. The sample was then glued to the tip of a stainless-steel tomography needle covered with UV light-sensitive adhesive (UHU BOOSTER LED Light®; UH48150). First, the sample was thinned down by laser ablation milling (using the 3D-Micromac microPREP™ PRO laser ablation system with 0.100 W, 20 kHz pulse frequency, 20 μm spot diameter and 1 μm pulse distance), removing the cathode catalyst layer. From the resulting pillar-like structure of the anode catalyst layer protruding from the top of the needle (~ 20 μm in height), the attached Nafion^TM^ NR212 membrane was ablated with the FEI Helios NanoLab 660 SEM/FIB Dual Beam system using Ga^+^ ions with 30 kV acceleration voltage and 0.1 nA beam current.

The 180° nano-CT tilt series (561 projections with 0.32° tilt increment, an image size of 1024 ∙ 1024 pixel and a pixel size of 16.09 nm) of the sample was acquired in the high resolution (HRES) PC mode (16 μm ∙ 16 μm field of view, ~ 50 nm spatial resolution) of the X-ray microscope with an illumination time of 80 s per frame. The tilt series was recorded using the native ZEISS microscope software (XMController and Scout&Scan). The resulting tilt series was aligned with the Active Motion Compensation tool^[13]^ implemented in the software XMController. For the final 3D reconstruction, a simultaneous iterative reconstruction technique (SIRT, 150 iterations)^[14]^ was employed and implemented as an in-house Python script based on the Astra toolbox^[15]^. The Arivis Vision4D software (v. 3.3) was used for 3D segmentation and visualization utilizing its random-forest-based machine learning implementation. The local pore sizes in the reconstructed, segmented, and binarized 3D volume were analyzed using maximum sphere inscription (MSI, cf. **Figure S13**d) implemented in ImageJ (FIJI)^[1]^ as local thickness and the size distribution was generated by the extracted data. The catalyst volume fraction was determined by Thermo Scientific^TM^ Avizo Software (2023.1).

*Focused ion beam scanning electron microscopy (FIB-SEM) and SEM cross-sectional imaging:* The morphology and thickness of the catalyst layers (CLs) were determined on pristine CCMs with three different loadings for the core-shell and Umicore catalyst, respectively. The CL morphology was examined by FIB-SEM. The measurements were performed using a Zeiss Crossbeam 540 FIB-SEM. For the FIB-sectioning, the stage was tilted to an angle of 54° from the horizontal. To shield the cross-section from unintended beam damage, platinum was deposited on the region of interest. The cross-section was excavated by ion milling. The front face was milled by applying a beam current of 15 nA and subsequently polished with a current of 7 nA. A secondary electron detector (SE2) was used to image the front face with an acceleration voltage of 3 kV and a probe current of 750 pA.

Cross-sectional imagining was employed on the same device to determine the thickness of the CLs. As preparation, CCM samples were embedded and polished as follows: A piece of ~ 1.5 ∙ 1 cm^2^ was cut out from the CCM sample with a scalpel. The cutout was sandwiched between two sheets of PTFE using plastic clips. The assembly was embedded in epoxy resin (EpoThin, Buehler Inc.), followed by grinding and polishing with a polishing machine (LaboPol-30, Struers GmbH) with a final grain size of 250 nm (DiaPro Nap ¼ µm, Struers GmbH). The embedded samples were imaged using the backscattered electron detector (BSD), exploiting mass contrast with an applied acceleration voltage of 20 kV and a probe current of 2 nA. The thickness was measured at five points of the cross section and the mean value with standard deviation is given.

*Electronic in-plane conductivity:* A four-electrode setup (Ossila B. V., Four-Point Probe T2001A3) was used to measure the in-plane electronic conductivity of the catalyst layers. The measurement was performed on the dried catalyst layers (area 5 cm^2^) coated onto a 50 µm thick PTFE sheet. All samples were measured at three points throughout the area and mean values with standard deviation are given. To avoid any influence on future measurements, electrodes that were tested in the four-electrode setup were not used for single-cell testing.

- 1. **Electrochemical characterization**

*Rotating disk electrode (RDE):* The electrochemical performance of the novel catalyst was compared to two commercial catalysts, IrO_x_ (Alfa Aesar Premion) and IrO_2_/TiO_2_ (Umicore Elyst Ir75 0480). For RDE measurements, a mirror-polished gold RDE electrode (5 mm diameter) was coated with catalyst ink. The catalyst ink was composed of iridium catalyst powder, ionomer (Nafion^TM^ 1100W, Sigma Aldrich), 1 mL of isopropanol (> 99.8%, Sigma-Aldrich), and 3 mL of DI water (18.2 MΩ cm). The amount of catalyst powder was chosen to yield a loading of 50 µg_Ir_ cm^-2^ on the RDE tip after drop-casting two droplets of 10 µL ink. The Nafion content was controlled to be 20 wt% of the solid content for the commercial catalysts and 10 wt% for the core-shell catalyst (a lower Nafion content was chosen because of the lower specific surface area). The ink was dispersed by 15 min of ultrasonication in an ultrasonic bath. A Pt wire electrode (99.99%, Pine Research) and a reversible hydrogen electrode (RHE Hydroflex, Gaskatel) were chosen as counter and reference electrode, respectively. All measurements were conducted at room temperature in 0.1 M HClO_4_ (diluted from 70%, EMSURE®, Sigma Aldrich), which was purged with Ar for 30 min before the measurement and blanketed with Ar during the measurement. The RDE tip was rotated at 1600 rpm in a commercial RDE setup (Pine Research, WaveVortex 10 Rotator). The measurement consisted of two techniques: First, five cyclic voltammograms at different scan rates (5, 10, 25, 50, and 100 mV s^‑1^) were recorded in the non‑faradaic region between 1.22 and 1.24 V vs. RHE. Secondly, three consecutive linear sweep voltammograms (LSVs) in the OER region between 1.1 and 1.8 V vs. RHE were performed with a scan rate of 5 mV s^-1^. Of these three LSVs, only the last one was taken for OER evaluation. All measurements were conducted three times on separate coatings for each catalyst ink. An approximate 80% iR correction was applied during all measurements as on-the-fly compensation *via* the potentiostat (Nordic Electrochemistry, ECi 210). The remaining resistance was continuously measured and subtracted from the data to give the *iR*-free potentials.

*Scanning flow cell (SFC) coupled to an inductively coupled plasma mass spectrometer (ICP‑MS):* The stability of the catalysts was measured with a scanning flow cell (SFC) coupled on‑line to an inductively coupled plasma mass spectrometer (ICP‑MS). A detailed description of the setup can be found elsewhere.^[16,17]^

Samples were prepared by drop-casting the catalysts on a polished gold plate (99.95%, Thermo Scientific) with a loading of 10 µg_Ir_ cm^-2^. Therefore, the catalysts were suspended in a H_2_O-based ink, containing 12.5% isopropanol (EMSURE®, Merck) and 20 wt% or 10 wt% Nafion (Nafion^TM^ 1100W, Sigma Aldrich) of the solid content for the commercial and core-shell catalysts, respectively. The ink was ultrasonicated for 15 min (4 s pulse, 2 s pause) with an ultrasonic horn. Then, the pH of the ink was adjusted to 11 by adding 1 M KOH (ROTI®METIC 99.98%, CarlRoth). To avoid sedimentation of the catalysts, the inks were sonicated for 5 additional minutes before drop-casting 0.2 µL on the gold plate. By using a vertical camera, the spots were positioned under the SFC opening.

Measurements were performed in Ar-purged 0.1 M HClO_4_, diluted from concentrated acid (70%, Suprapur, CarlRoth) with DI water (18.2 MΩ cm) with a Gamry Reference 620 potentiostat (Gamry Instruments). A glassy carbon rod (⌀ = 6 mm, HTW Hochtemperatur-Werkstoffe) was used as the counter electrode, and a double‑junction silver-silver chloride electrode (Metrohm, inner junction: 3 M KCl (Merck), outer junction: 0.1 M HClO_4_) as the reference electrode. All potentials are reported versus the reversible hydrogen electrode (RHE), which was measured daily before measurements using a Pt‑wire (⌀ = 0.5 mm, 99.99%, MaTeck) in hydrogen-saturated electrolyte. The following electrochemical protocol was used: Hold at E = 1.1 V for 360 s. Then, an LSV from 1.1 – 1.65 V with a scan rate of 5 mV s^-1^, followed by a hold at 1.1 V before a chronopotentiometry for 300 s at 100 mA mg_Ir_^-1^ and the final hold for 360 s at 1.1 V. To ensure reproducibility and statistics, each measurement was performed three times on three different drop-cast spots.

The SFC was coupled on-line to an ICP-MS (Agilent 7900, Agilent Technologies). Calibration was performed daily with standard solutions (Certipur ICP-MS Standard, Merck). The flow rate of the electrolyte from the SFC to the ICP-MS was measured daily. Before entering the ICP-MS, the sample was mixed with an internal standard solution (^187^Re for ^193^Ir in electrolyte prepared from Certipur ICP-MS Standard, Merck).

- 1. **Statistical Analysis**

For STEM investigations, the micrographs have been processed using Velox (v. 3.9.0) and FIJI.^[1]^ The structure sizes, as determined *via* STEM, are reported as mean value ± standard deviation. The number of measurements (sample size *n*) is not given in the main manuscript to facilitate the reading; instead they are listed here: TiO_2_ support particles: 750 ± 360 nm (*n* = 51 measurements); IrO_x_ shell: 2.1 ± 0.4 nm (*n* = 56 measurements); additional Ir nanoparticles: 34 ± 9 nm (*n* = 323 measurements); Seeds after photodeposition: 0.9 ± 0.2 nm (*n* = 54 measurements); Seeds after photodeposition and IPA reduction: 1.4 ± 0.4 nm (*n* = 22 measurements).

**2. Supporting Figures**


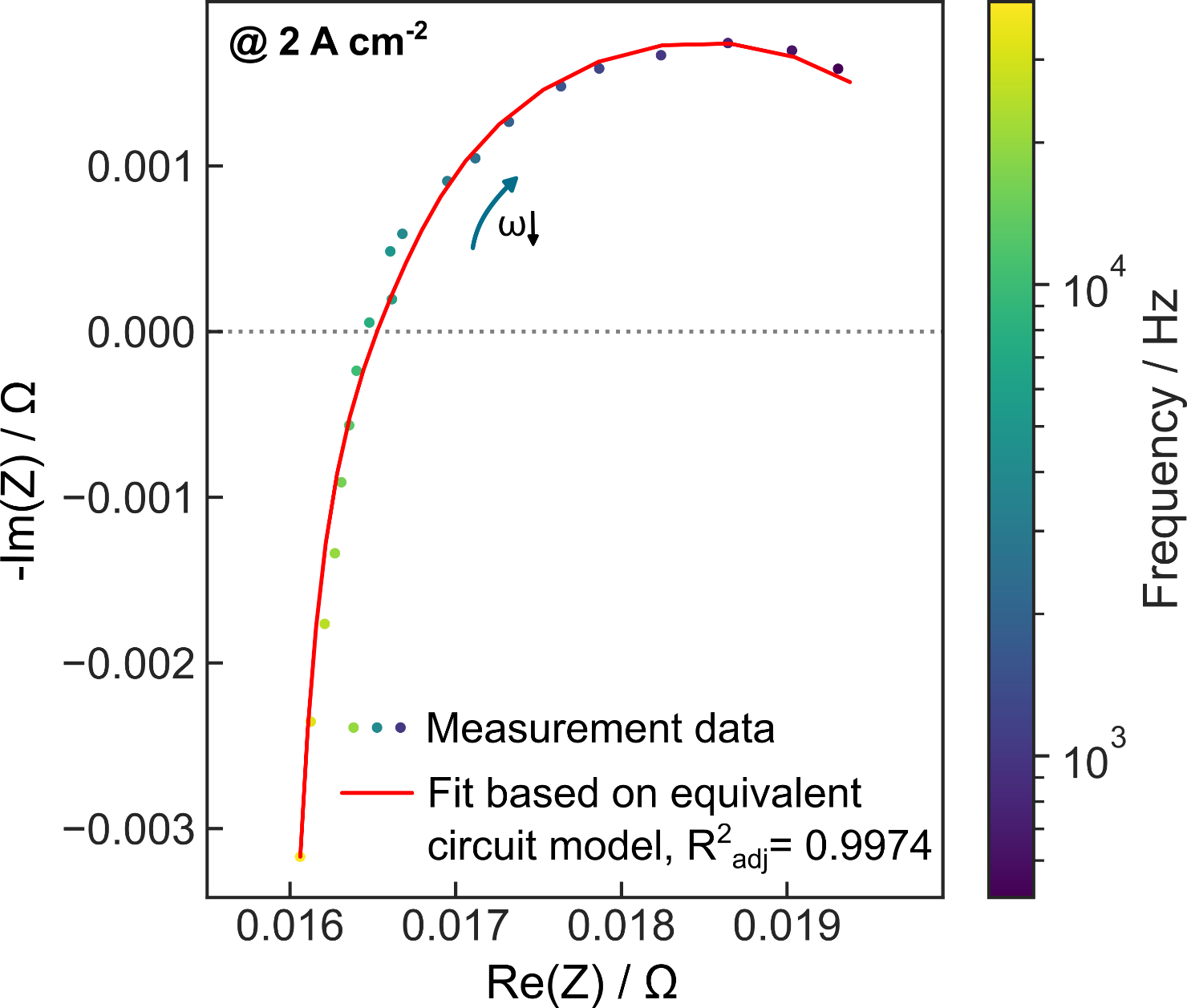


**Figure S1.** Exemplary Nyquist plot along with equivalent circuit model fit for determining the HFR values. The Nyquist plot is measured with galvanostatic electrochemical impedance spectroscopy at 2 A cm^-2^ as described in the experimental section. A CCM with Nafion NR212, 0.3 mg_Ir_ cm^-2^ (Umicore Elyst Ir75) on the anode and 0.2 mg_Pt_ cm^-2^ Pt/C on the cathode was used. Only fitted data points in the frequency interval of 50 – 0.5 kHz are shown. Adjusted R^2^ (R^2^_adj_ ≥ 0.99) is used as a quality indicator for the fit. The equivalent circuit model used for fitting comprises an inductance, a resistor, and a transmission line model in series.^[18,19]^


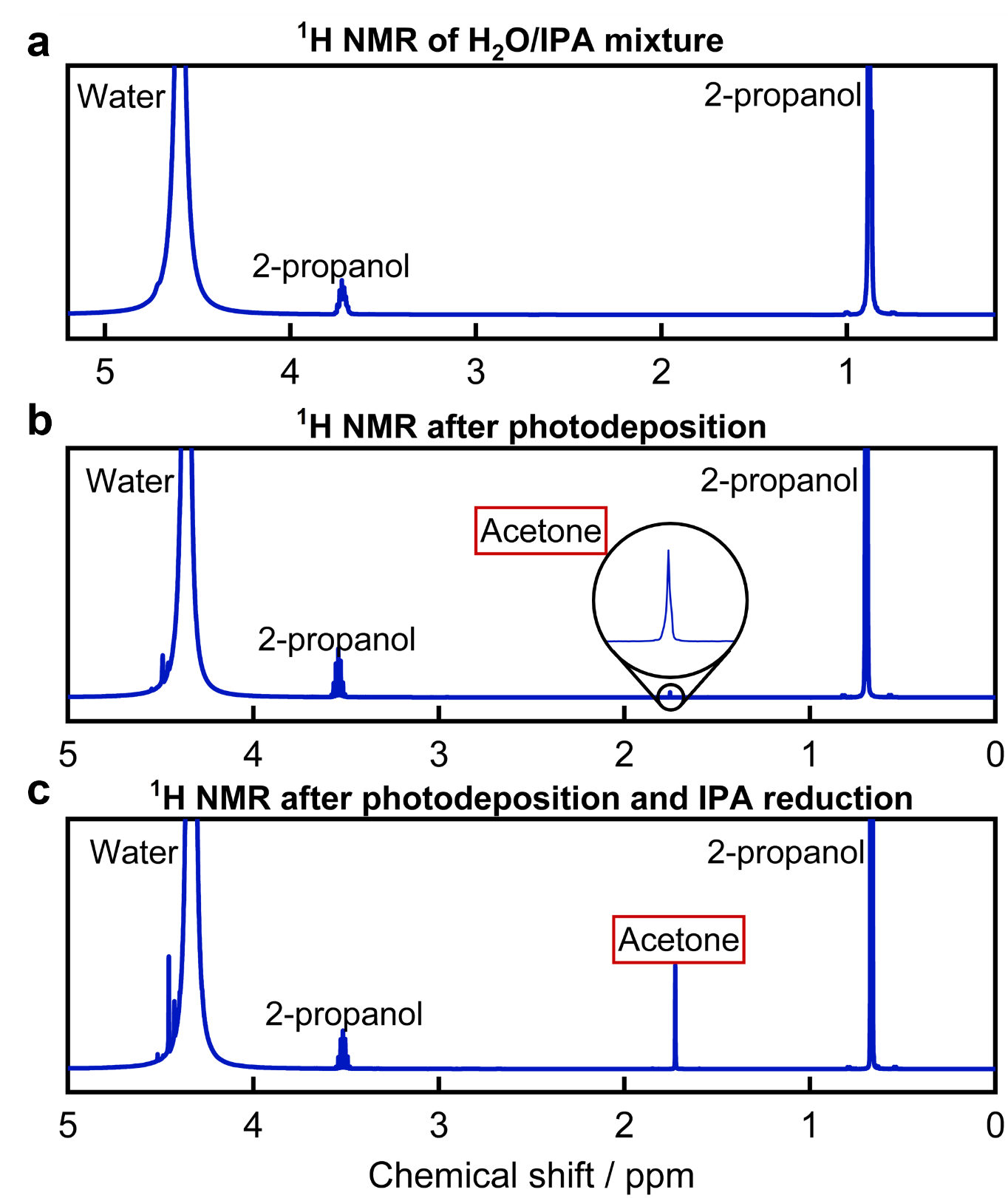


**Figure S2.** ^1^H NMR of (a) H_2_O/IPA mixture, (b) the reaction solution after the photodeposition step, and (c) the reaction solution after both photodeposition and IPA reduction. The increasing acetone signal evidences IPA oxidation and therefore proves its utilization as both hole scavenger and chemical reduction agent during the iridium deposition steps.


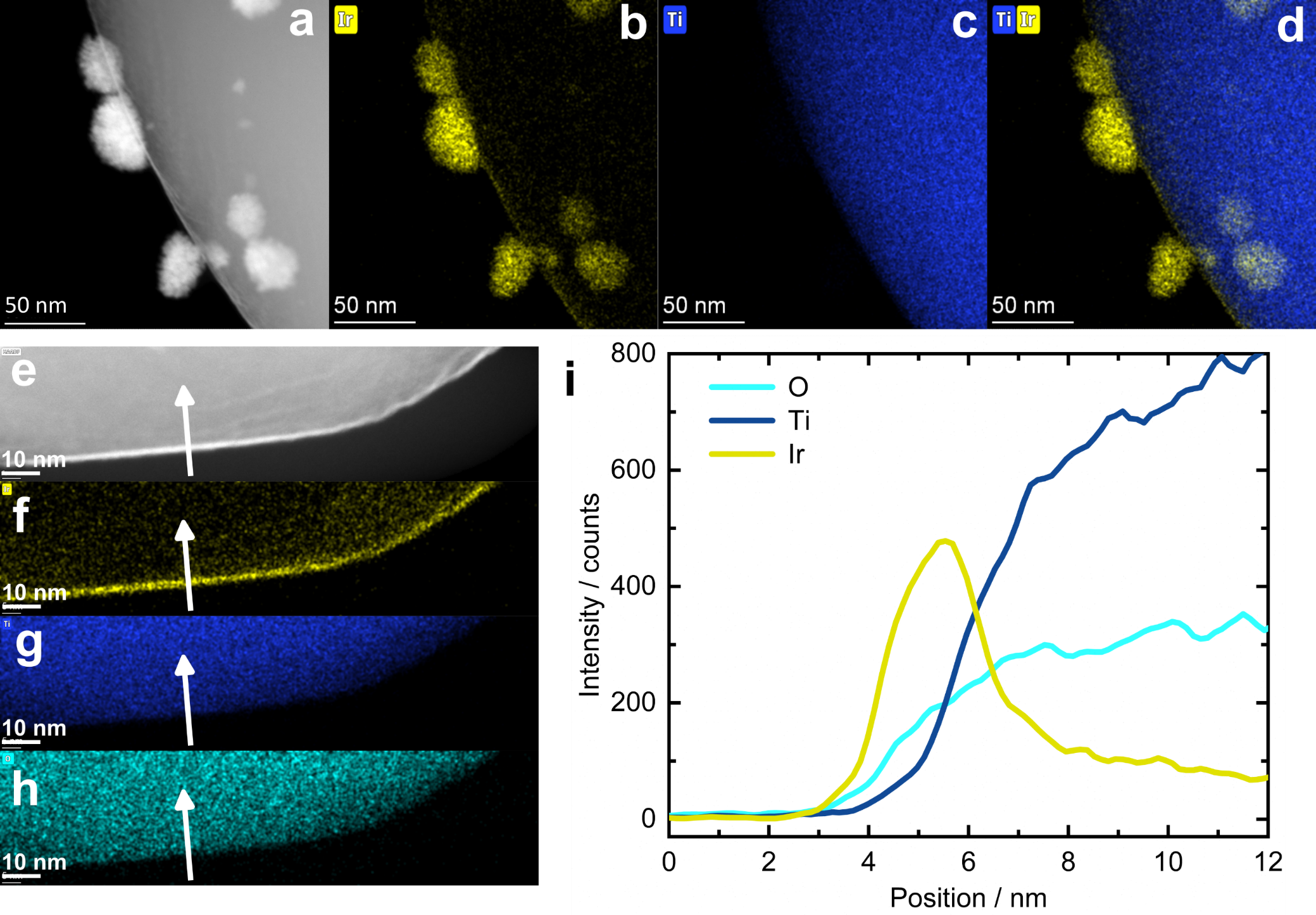


**Figure S3.** (a) – (h) HAADF-STEM/EDX spectrum images of the synthesized TiO_2_@IrO_x_ core-shell catalyst. (i) EDX line profile of the marked region in images (e) – (h).


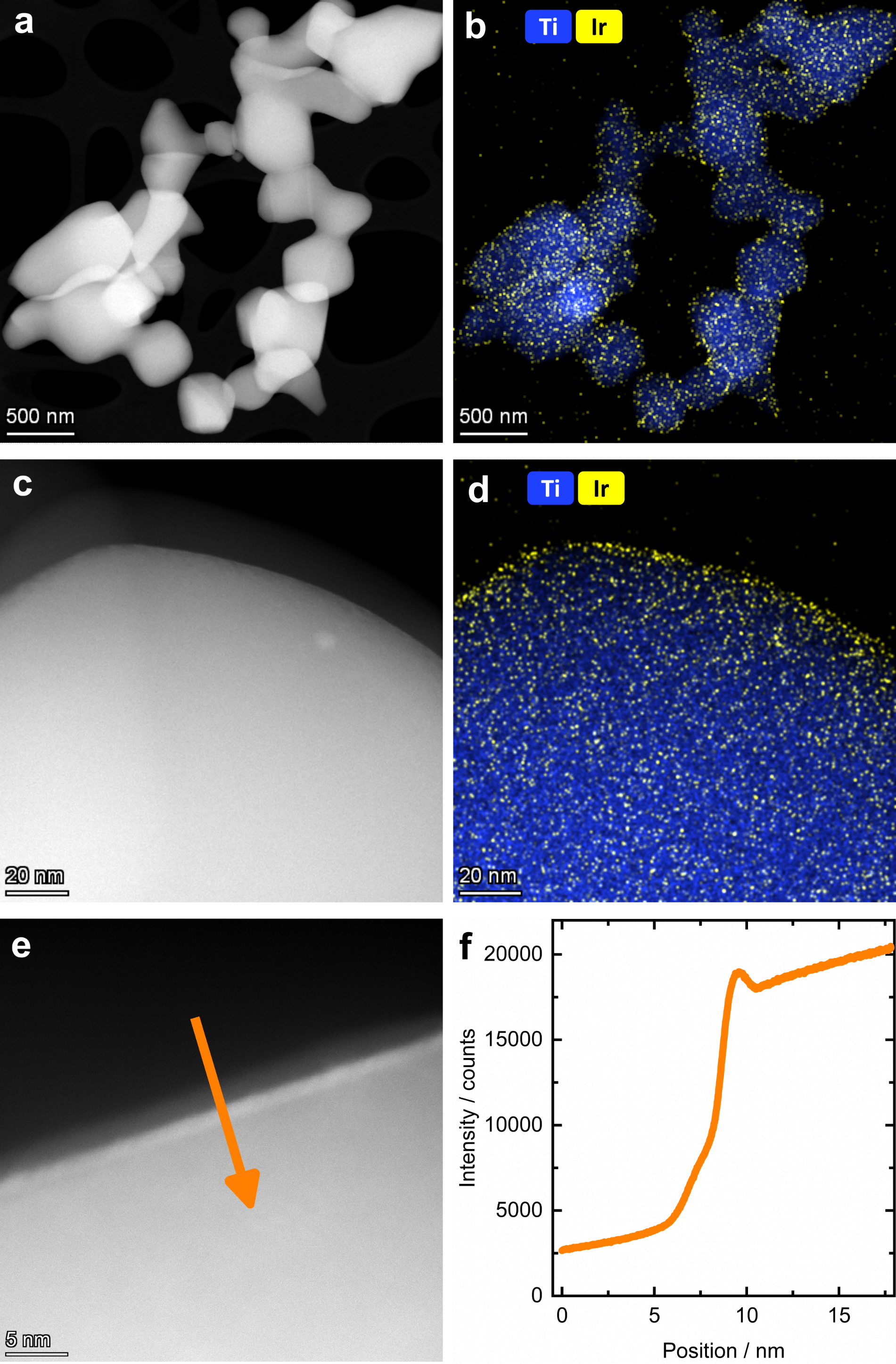


**Figure S4.** (a) – (e) HAADF-STEM/EDX spectrum images of the TiO_2_@IrO_x_ catalyst with only the nanometer-thin IrO_x_ shell (no additional Ir nanoparticles). This structure is achieved by only performing the photodeposition synthesis step followed by annealing (the IPA reduction step is omitted in this case). (f) HAADF line profile of the marked region in image (e).


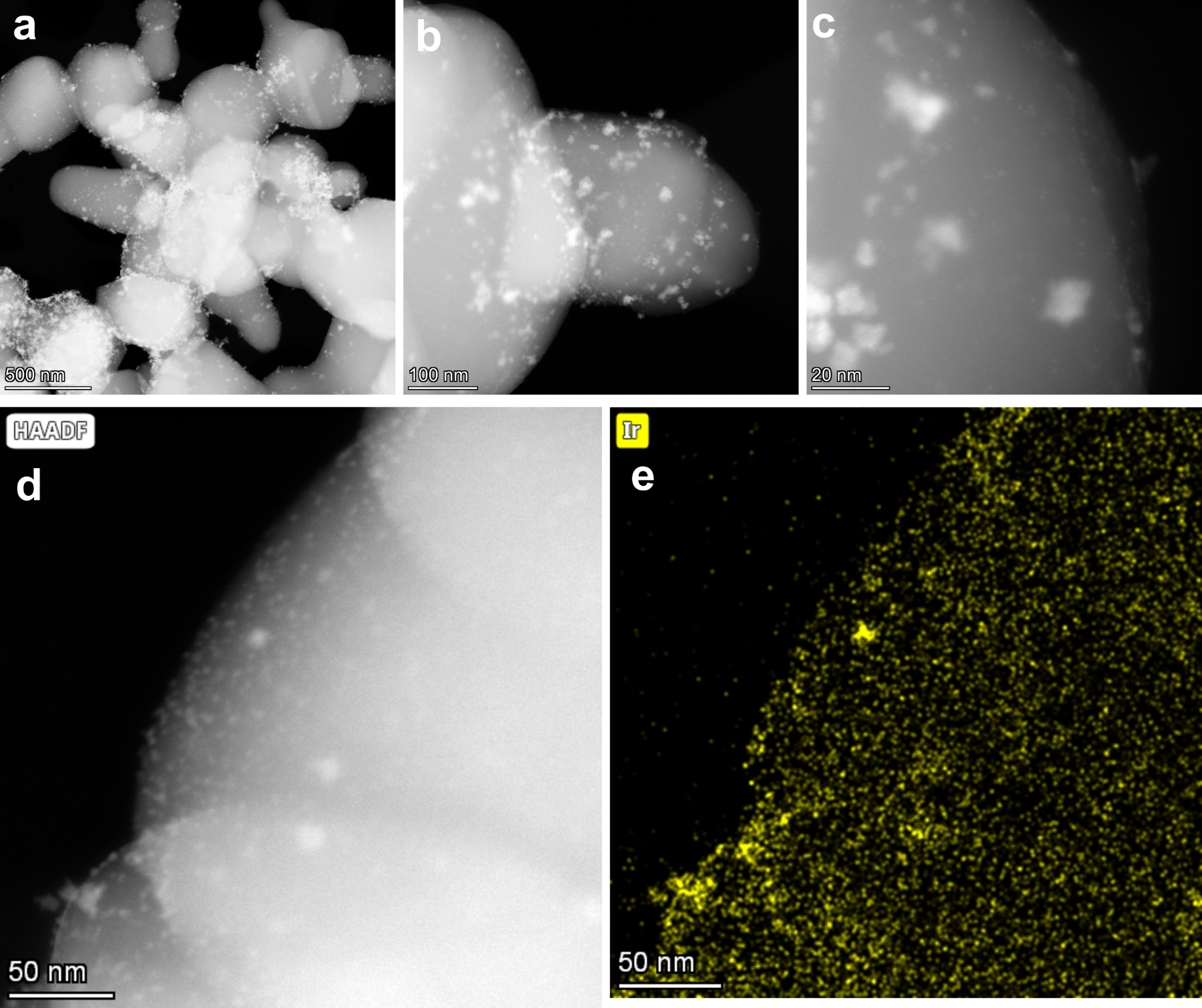


**Figure S5.** (a) – (e) HAADF-STEM/EDX spectrum images of the IrO_x_/TiO_2_ catalyst without the nanometer-thin IrO_x_ shell. This structure is achieved by only performing the IPA reduction synthesis step followed by annealing (the photodeposition step is omitted in this case).


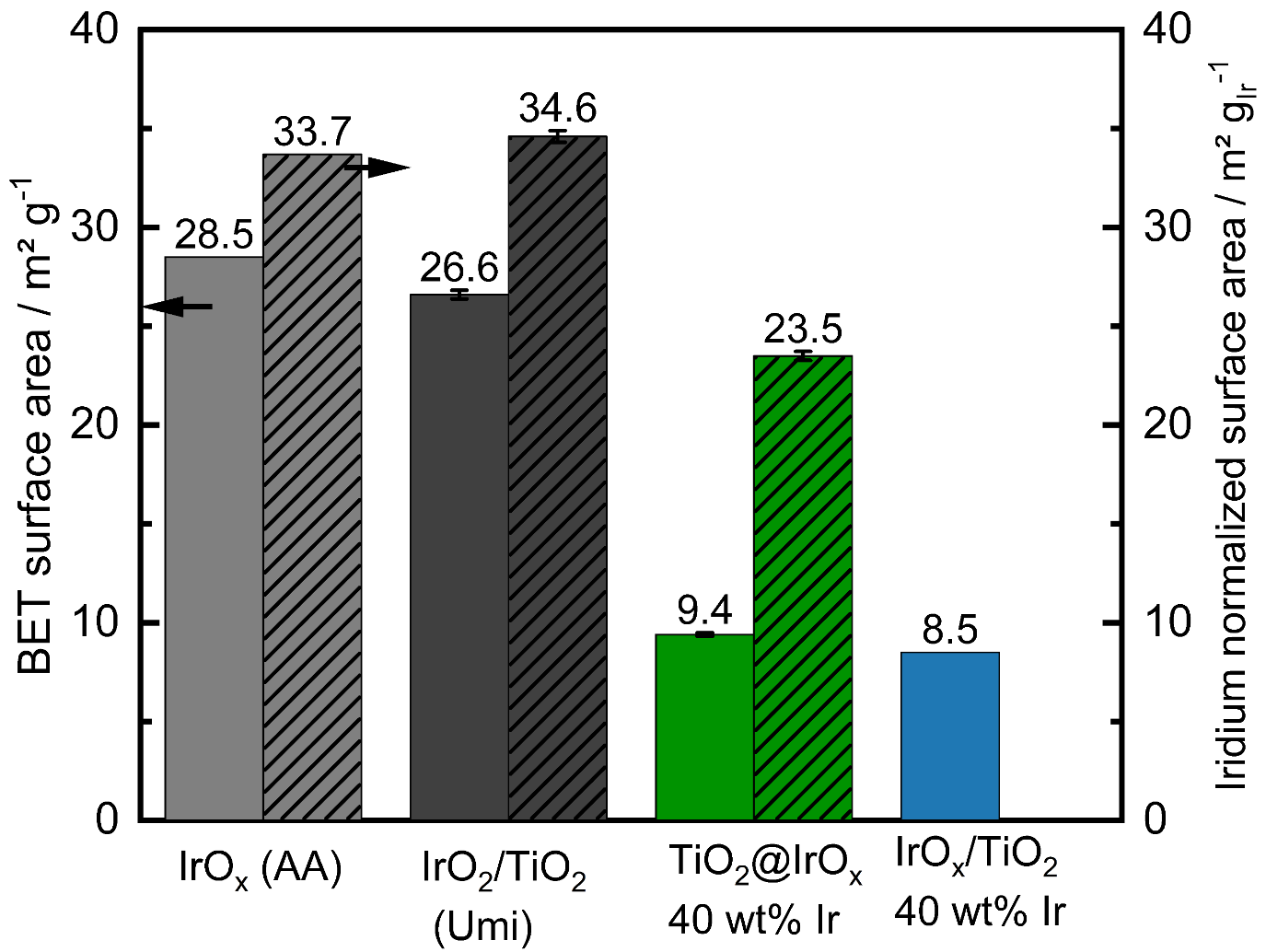


**Figure S6.** BET surface areas (left axis) and iridium-mass normalized surface areas (right axis) of two commercial catalysts (Alfa Aesar Premion and Umicore Elyst Ir75 0480), the novel TiO_2_@IrO_x_ core-shell catalyst, and the IrO_x_/TiO_2_ (no shell) catalyst. For the calculation of the iridium normalized surface areas, iridium contents of 84.5 wt% (AA), 75 wt% (Umi), and 40 wt% (core-shell) were used. For the IrO_x_/TiO_2_ catalyst, TiO_2_-surface area is exposed (cf. Figure S5) and therefore, the iridium normalized surface area cannot be calculated from the measured BET surface area.


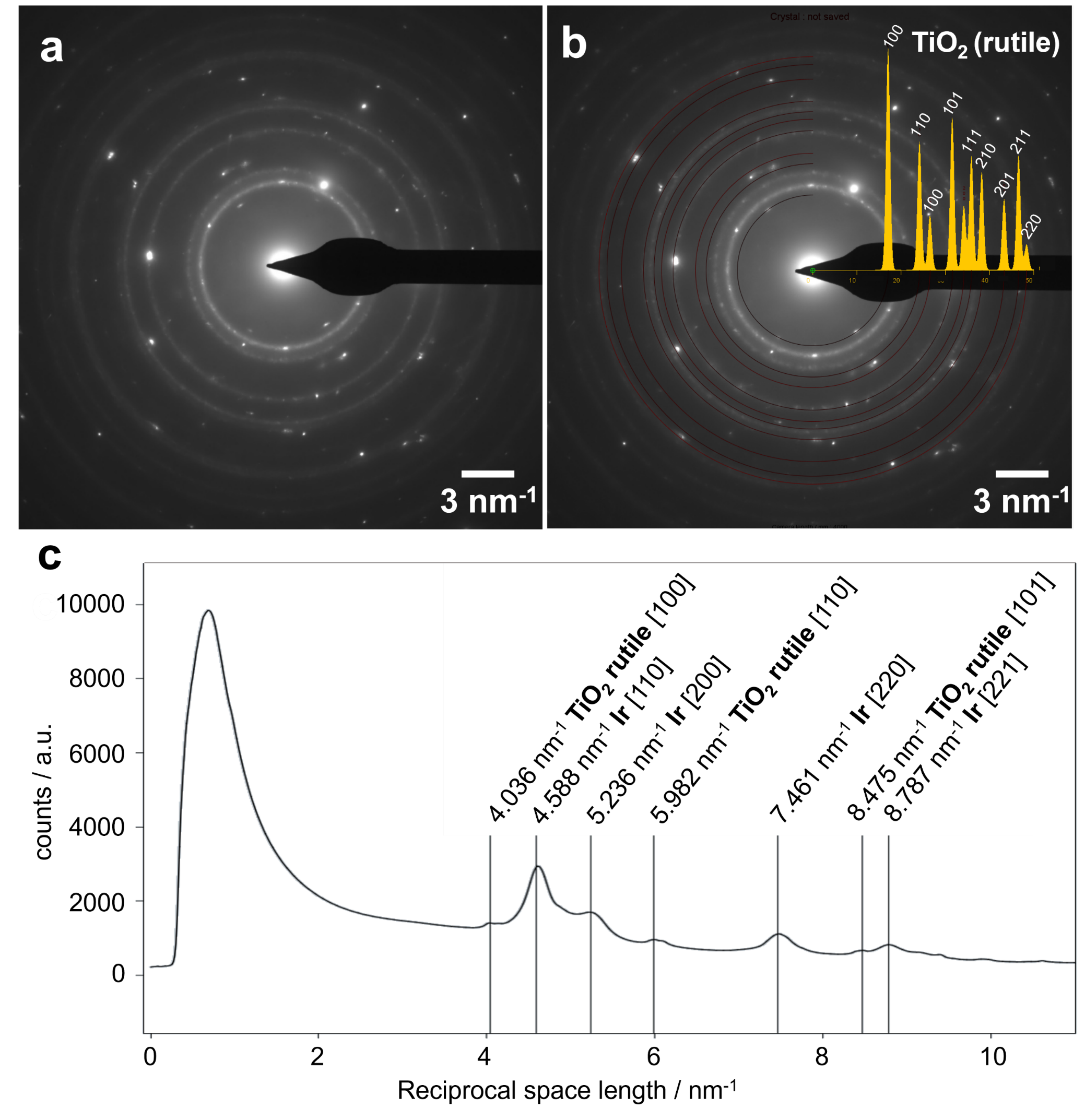


**Figure S7.** (a) Electron diffraction (ED) pattern of the lamella sample (cf. Figure 3c and d in main manuscript) (b) Yellow overlay: expected rotationally averaged peaks of arbitrarily oriented rutile TiO_2_. (c) ED spectrum with expected peaks of rutile TiO_2_ and fcc iridium.


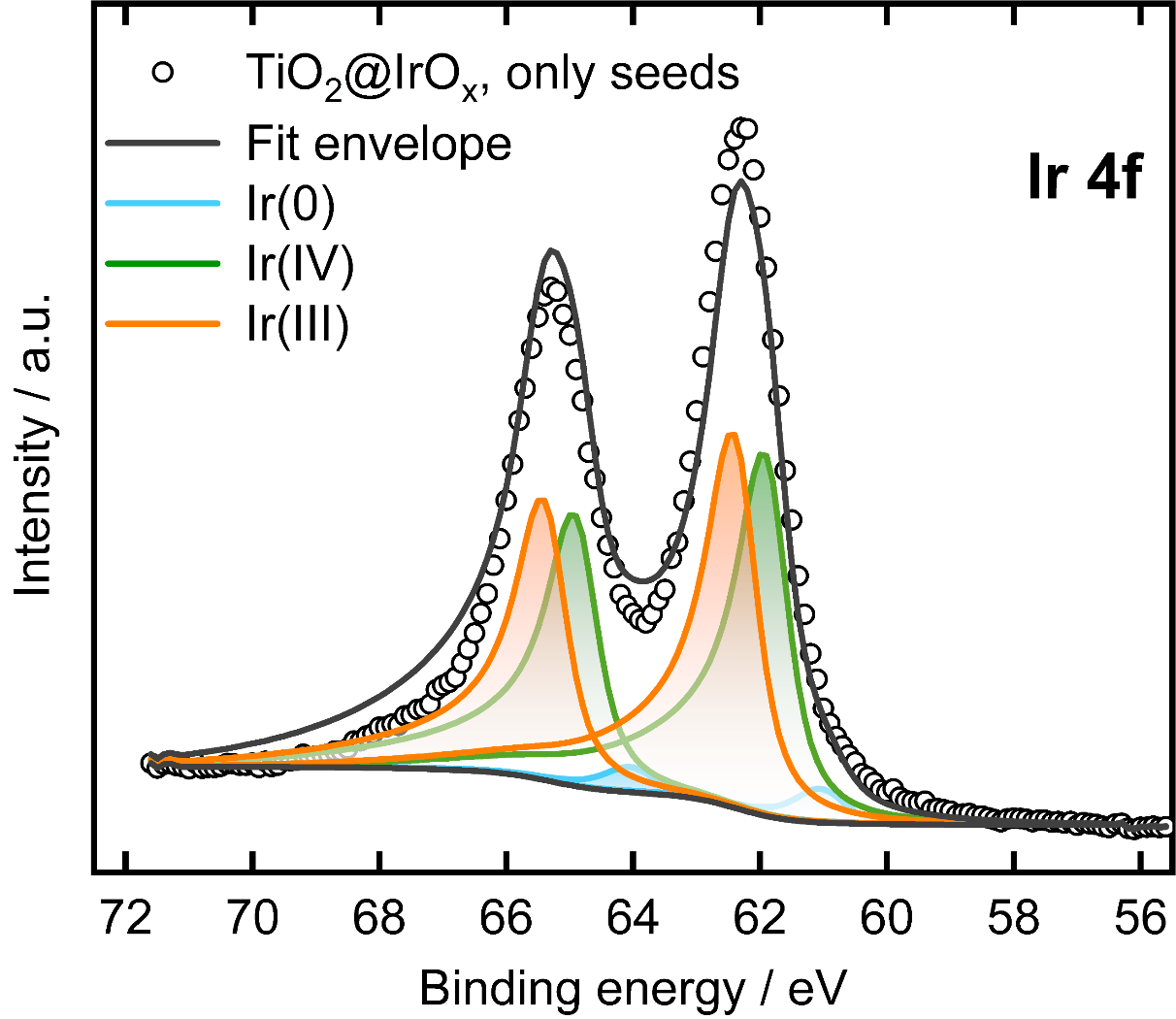


**Figure S8.** Ir 4f XPS spectrum and deconvolution fitting of the iridium seeds on the TiO_2_ support (cf. STEM images in Figure 2a and b).


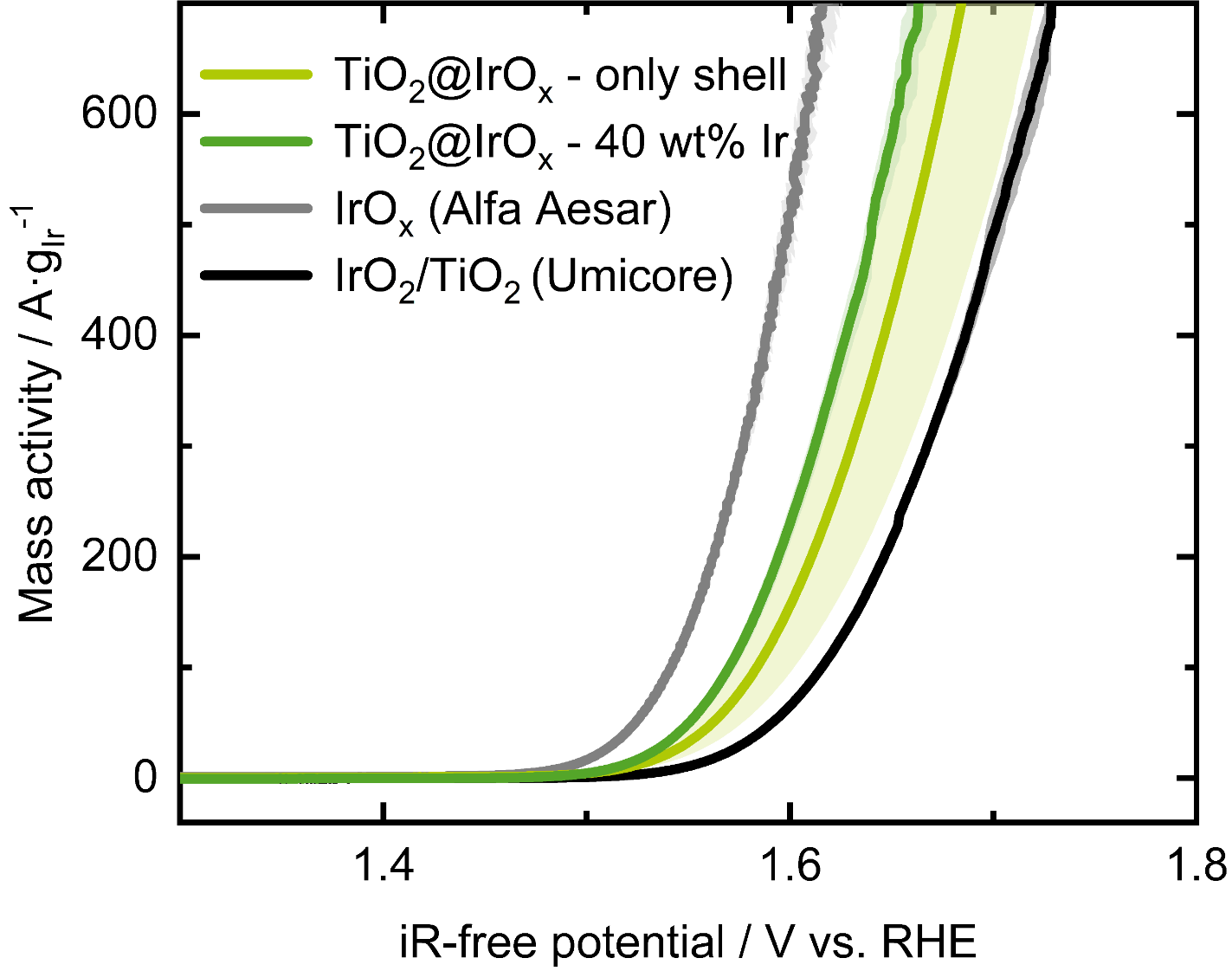


**Figure S9.** Iridium-mass specific LSVs of the TiO_2_@IrO_x_ core-shell catalyst after photodeposition and annealing at 350 °C (“only shell”, 2.7 ± 0.6 wt% Ir, cf. TEM image in Figure S4), TiO_2_@IrO_x_ (40 wt% Ir), IrO_x_ (Alfa Aesar Premion) and IrO_2_/TiO_2_ (Umicore Elyst Ir75). Shaded areas in the LSVs correspond to the standard deviation of at least three measurements. LSVs were conducted in an RDE setup in Ar-purged 0.1 M HClO_4_ at a loading of 50 µg_Ir_ cm^-2^ for the TiO_2_@IrO_x_ (40 wt% Ir) catalyst and the reference samples and 10 µg_Ir_ cm^-2^ for the “only‑shell” specimen.


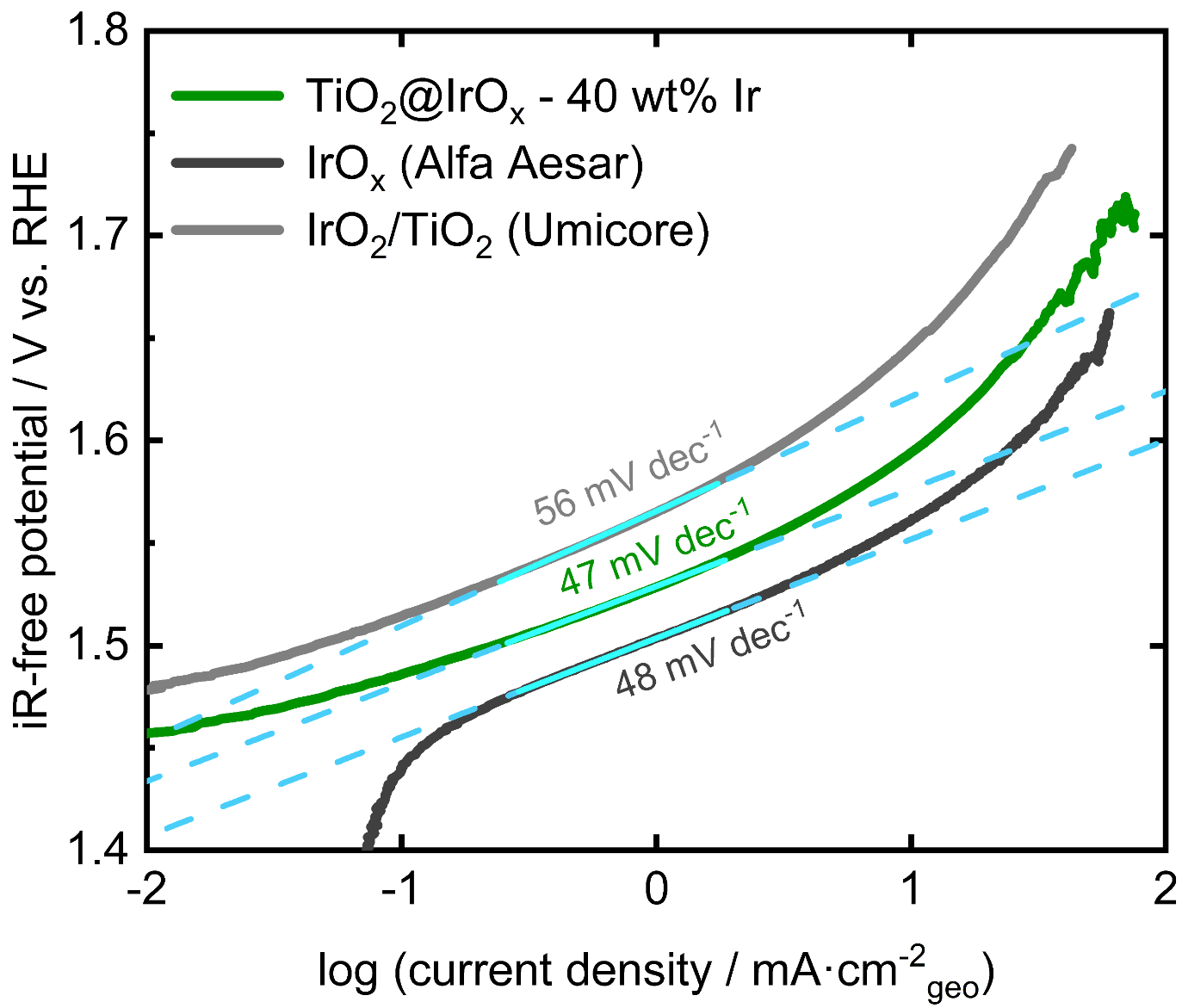


**Figure S10.** Tafel slope analysis of the TiO_2_@IrO_x_ core-shell catalyst and the two commercial reference catalysts (Alfa Aesar Premion and Umicore Elyst Ir75). The measurements were conducted in an RDE setup in Ar-purged 0.1 M HClO_4_ at a loading of 50 µg_Ir_ cm^-2^.


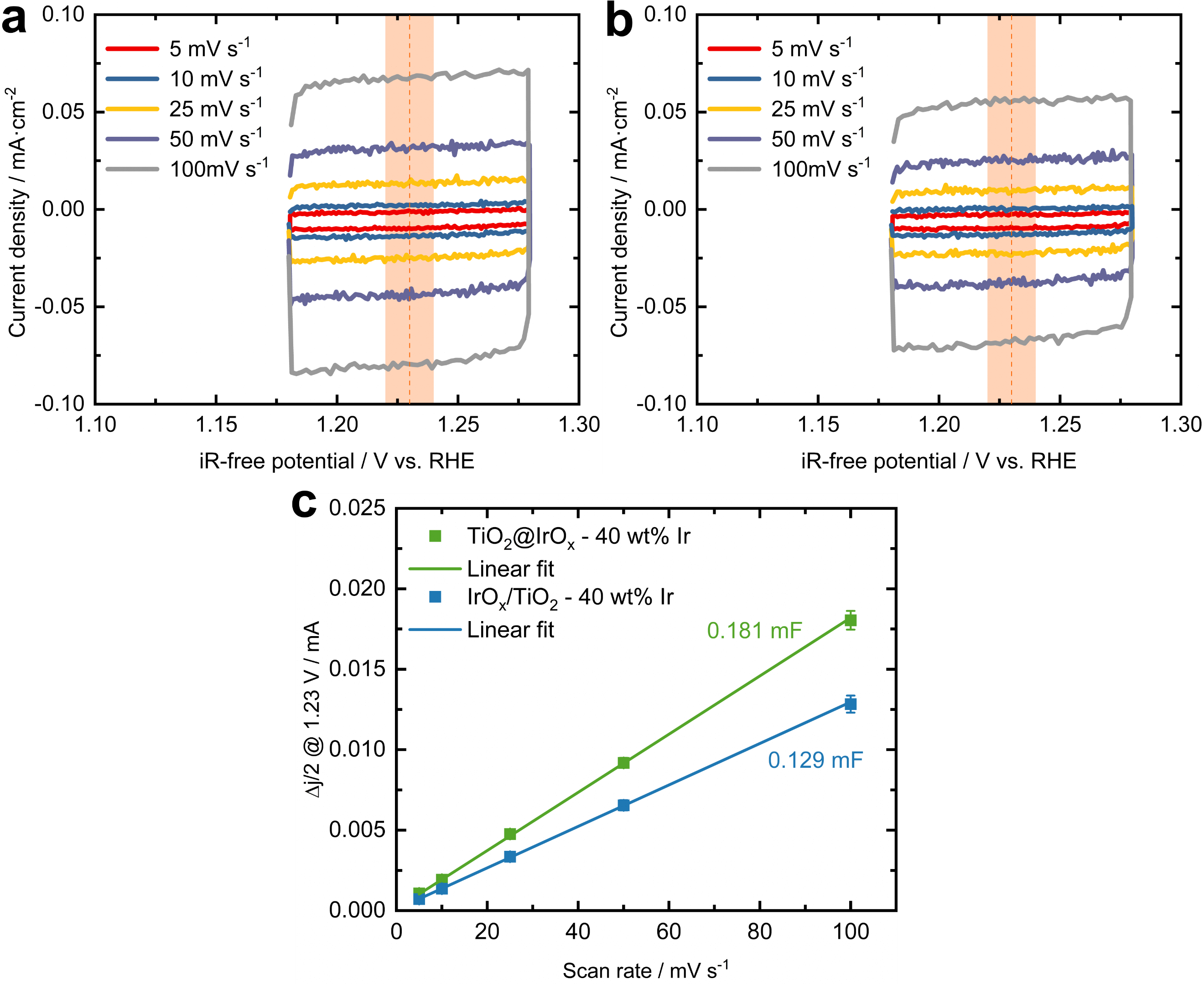


**Figure S11.** Cyclic voltammograms at different scan rates in the non-faradaic region of the (a) TiO_2_@IrO_x_ core-shell and (b) IrO_x_/TiO_2_ (without shell) catalysts. The pseudocapacitive currents were averaged between 1.22 and 1.24 V vs. RHE (marked region) for three samples each and are plotted over the scan rate in (c). A linear fit yields the pseudocapacitances as an approximation of the electrochemically active surface area (ECSA). All measurements were conducted in an RDE setup in Ar-purged 0.1 M HClO_4_ at a loading of 50 µg_Ir_ cm^-2^.


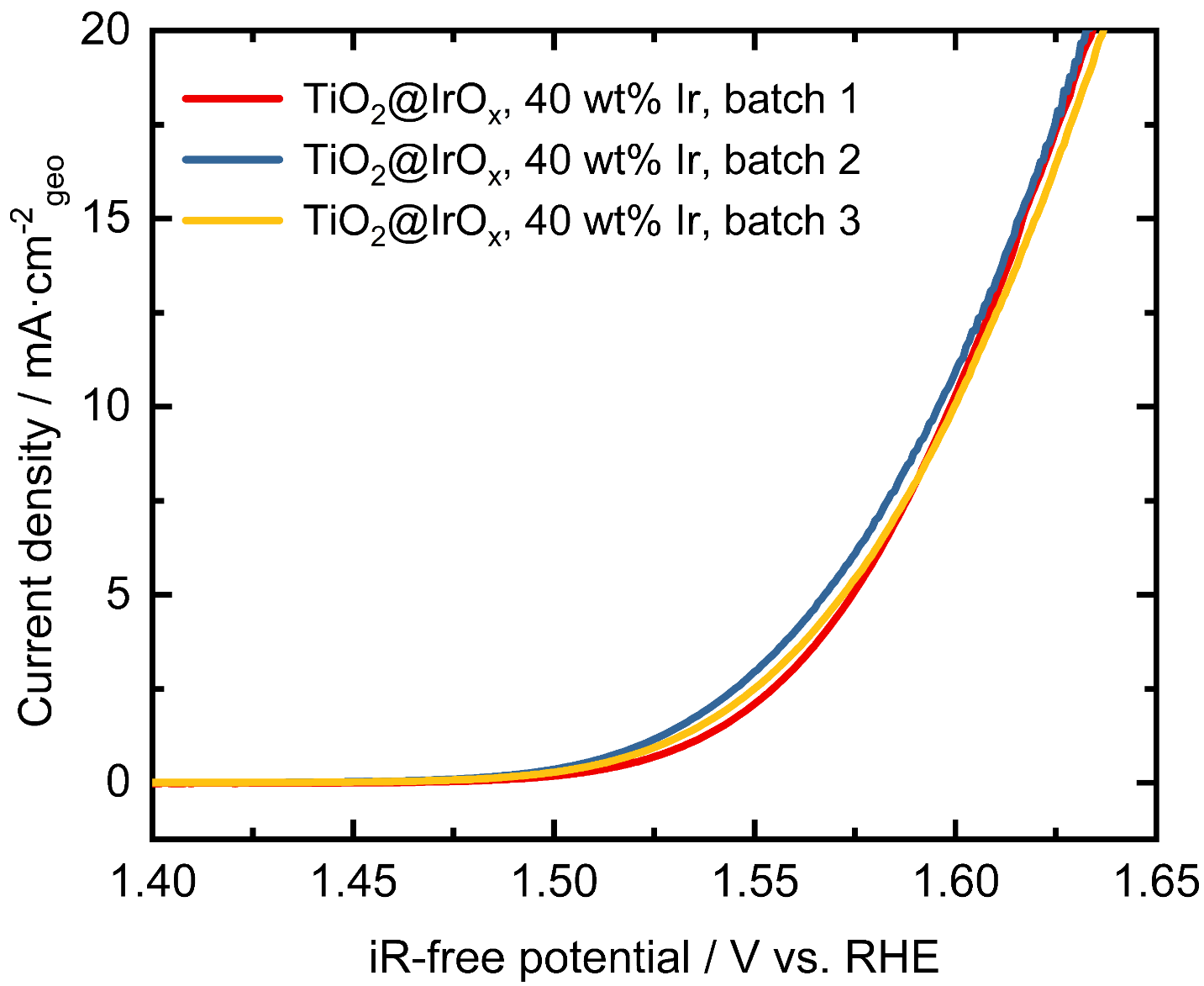


**Figure S12.** The OER activities of three different synthesis batches of the TiO_2_@IrO_x_ core-shell catalyst show the reproducibility of the synthesis method. Deviations at higher current densities are attributed to bubble formation. All measurements were conducted in an RDE setup in Ar-purged 0.1 M HClO_4_ at a loading of 50 µg_Ir_ cm^-2^.

Tips for reproducing the synthesis and measurement results of TiO_2_@IrO_x_ core-shell particles:

- To guarantee the transmission of UV-C radiation, quartz glass should be used. We observed that irradiation from the side (with the reaction solution in a quartz flask) can lead to iridium deposition on the inside of the glass. This deposited iridium hinders the transmission of UV-C radiation and should be avoided. Therefore, an irradiation from the top was chosen, with the reaction solution in a PTFE beaker and a quartz glass slide on top. This quartz glass slide prevents solvent evaporation and allows the transmission of the radiation. Note that in this case, the quartz glass is not in contact with the reaction solution.
- For RDE measurements, it was observed that a Nafion content of 10 wt% of the solid content (found as optimum value in literature^[20]^) leads to homogeneous coatings and high OER activities. In contrast, a Nafion content of 20 wt% also leads to homogeneous coatings but significantly reduced OER activity. This is attributed to the lower surface area of the TiO_2_@IrO_x_ core-shell particles in this work. For the commercial catalysts Umicore Elyst Ir75 and Alfa Aesar Premion, a Nafion content of 10 wt% yielded inhomogeneous catalyst layers and 20 wt% Nafion was needed to achieve homogeneous coatings and good performance. We attribute this behavior to the drop-casting method and emphasize that different optimal Nafion contents are expected for other coating techniques.


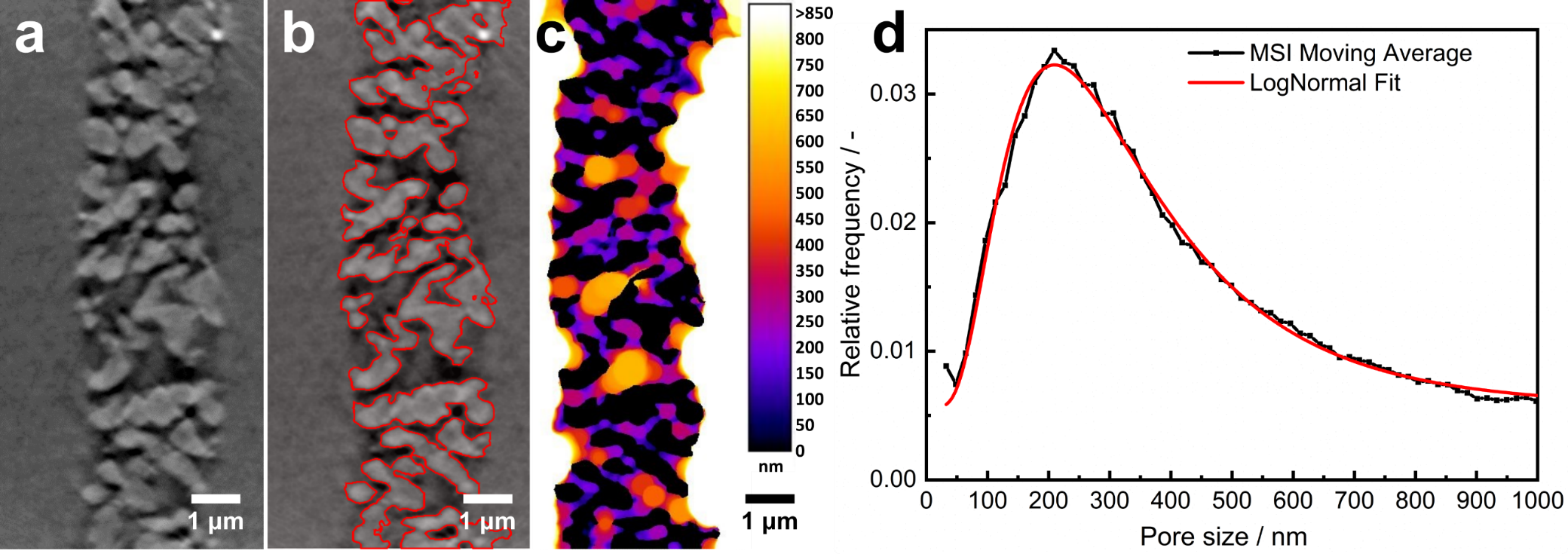


**Figure S13.** Single exemplary virtual slice through the 3D high-resolution phase-contrast nano-CT reconstruction displayed as (a) 16-bit greyscale image (see also video V.2), (b) segmentation of solid structure (brighter contrast) with outlines indicated in red, and (c) maximum sphere inscription (MSI) pore size analysis with color-coded local pore diameters (see also video V.4). (d) Resulting pore size distribution (pores include void volume and ionomer film due to method) of the MSI analysis (plotted with moving average of 10 data points width) over the entire reconstructed volume. The fit is done with a log-normal distribution with a modal value of 209.6 nm and a mean value of 360.1 nm. The extracted catalyst volume fraction is 31.4%.

**2.1 Electronic in-plane conductivity of catalyst layers (CL)**

The in-plane conductivities of pristine and dry CLs were measured for the core-shell and the Umicore catalyst. The results are shown as a function of varying iridium loadings in **Figure S14**.

The absolute values of powder and in-plane conductivity cannot be directly compared due to the difference in pallet and CL thickness. Hence, the thickness-normalized in-plane conductivity gives more insight. Compared to the powder conductivity values, where the Umicore catalyst has 14‑fold higher conductivity, the difference in normalized in-plane conductivity between TiO_2_@IrO_x_ and IrO_2_/TiO_2_ is less pronounced with a factor of ~ 6 (core-shell 2.5 ± 0.5 S cm^-1^ vs. 14.4 ± 0.4 S cm^-1^). The difference in conductivity between TiO_2_@IrO_x_ and IrO_2_/TiO_2_ would be expected to remain in the same order of magnitude for the normalized in-plane and powder conductivity. The reason for the deviation could be found in the structural parameters of the layer but is up to now unknown and needs further investigation. Nevertheless, it can be concluded that incorporated in a CL, the intrinsic conductivity of the particles is not the only relevant factor, and the suitability of a catalyst should be evaluated by a combination of *in situ* and *ex situ* measurements.


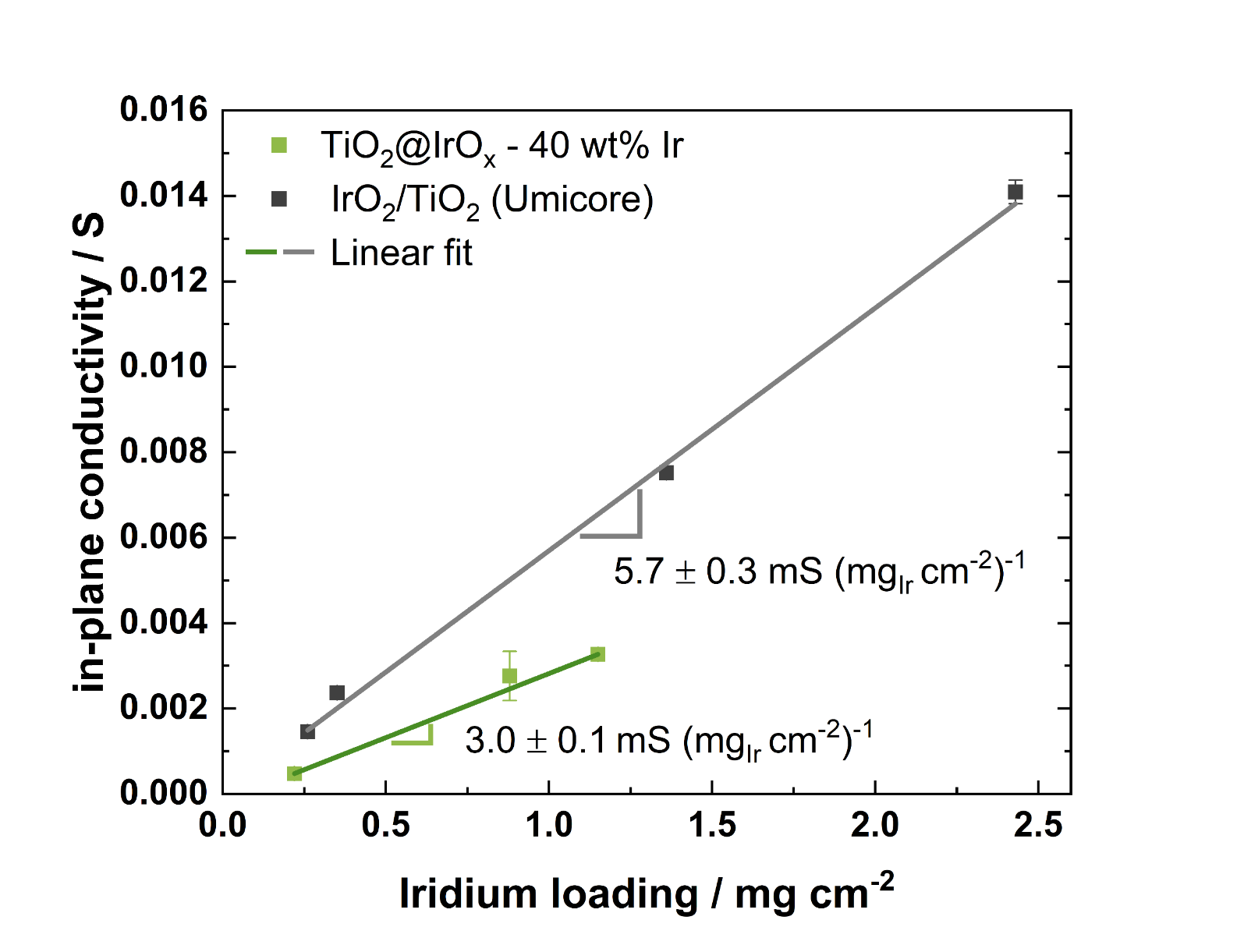


**Figure S14.** In-plane conductivity measurements of anode electrodes coated onto PTFE foil. The measurements are conducted at ambient conditions. The graph shows mean values from three spots on each electrode ± standard deviation. The thickness-normalized in-plane conductivities yield 2.5 ± 0.5 S cm^-1^ and 14.4 ± 0.4 S cm^-1^ for the core-shell and the Umicore catalyst, respectively. Thickness normalized in-plane conductivities of the two shown catalysts are determined based on the thickness factor in Figure 6a.


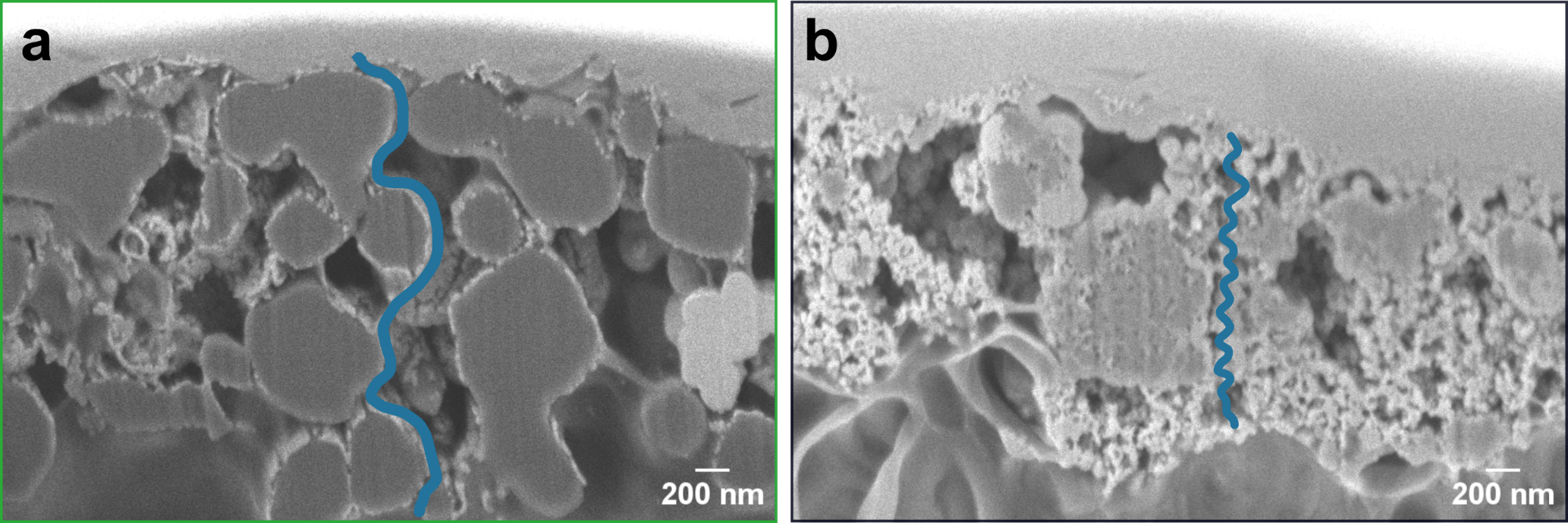


**Figure S15.** Ionic percolation pathway in (a) TiO_2_@IrO_x_ core-shell catalyst layer and (b) IrO_2_/TiO_2_ Umicore catalyst layer.

**2.2 Stability testing**

To investigate the stability of the catalyst, SFC-ICP-MS measurements were conducted using the protocol shown in **Figure S16**a. As a descriptor for the stability of an OER catalyst, the S-number is commonly used, which is defined as the number of evolved oxygen molecules per dissolved iridium atom.^[21]^ Therefore, the number of oxygen molecules is calculated *via* the integrated current (shaded area in the upper panel in Figure S16a) using Faraday’s law and divided by the integrated iridium dissolution during the current hold (shaded area in the lower panel in Figure S16a). The S‑numbers of the different catalysts are plotted in Figure S16b.

It can be clearly seen that the commercial catalyst from Umicore exhibits the highest dissolution stability with an S-number of (5.9 ± 1.6) ·10^5^, which is once more attributed to the complete oxidation to rutile IrO_2._ Promisingly, the stability of the TiO_2_@IrO_x_ core-shell catalyst is similar to the Alfa Aesar reference within the error margin (S-numbers: (1.19 ± 0.13) ·10^5^ vs. (1.47 ± 0.19) ·10^5^). The novel catalyst without the shell (IrO_x_/TiO_2_) exhibits the lowest stability with an S-number of (9.5 ± 0.9) ·10^4^. However, it is on the same order of magnitude as the core-shell sample, so no conclusion about the stability of the thin shell can be drawn. Finally, we emphasize that S-numbers provide an initial insight regarding catalyst stability. However, since they are determined in aqueous model systems, the stability in a PEMWE cell might differ and therefore stability tests on a single-cell level were performed.^[22,23]^


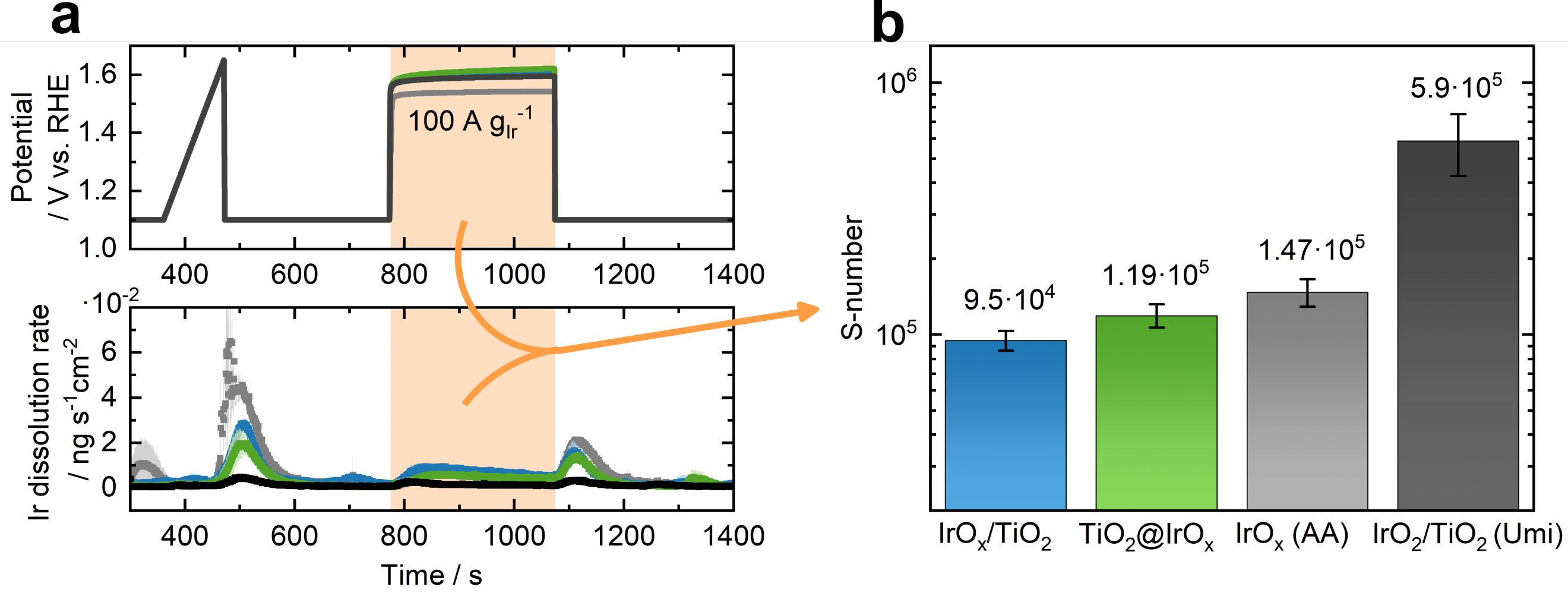


**Figure S16.** (a) Electrochemical protocol and time-correlated iridium dissolution rate, measured in an SFC-ICP-MS setup in Ar-purged 0.1 M HClO_4_ at a loading of 10 µg_Ir_ cm^-2^. (b) S-numbers calculated from SFC-ICP-MS measurements. Error bars depict the standard deviation of three measurements.

The stability of the core-shell and the reference catalyst were examined in single-cell testing in addition to the SFC-ICP-MS measurement. One test each was conducted over 200 h at a constant current density of 2 A cm^-2^ for a CCM with core-shell and reference catalyst at low loadings. The measured cell potential response is shown in **Figure S17**a and b for the two different catalysts, respectively. Two interruptions of the measurements for the core-shell catalyst and one interruption for the reference are marked in the graph by red diamonds. For the measurements with the core-shell CL, there is a first unscheduled shut-down after 36 h of current hold, which was caused by a failure of the nitrogen supply needed for a safe test bench operation. The current supply had to be stopped while heating and water supply were continued. Upon restart, a slight decrease in cell potential is observed, which can be assigned to the interruption. This effect has already been shown in other works where a shut-down caused a reduction of the catalyst to hydrous iridium oxide, which was then, at restart, re-oxidized to iridium oxide, which influences the OER activity.^[24]^ The second interruption of constant current at 110 h was planned, and nitrogen purging, heating, and water feed were continued.

Over the course of 200 h, the cell potential of the core-shell CL increases by 50 mV, whereas the Umicore catalyst only increases by 7 mV. However, the increase is not steady over 200 h but can be separated into several parts with different slopes. The slopes were determined by a linear regression in the last 20 h of the different regions. For the core-shell catalyst, a degradation of 0.17 mV h^-1^ was measured in the first part, followed by a sequence with a higher degradation of 0.44 mV h^‑1^. After around 130 h current hold, the degradation slows down to 0.25 mV h^-1^.


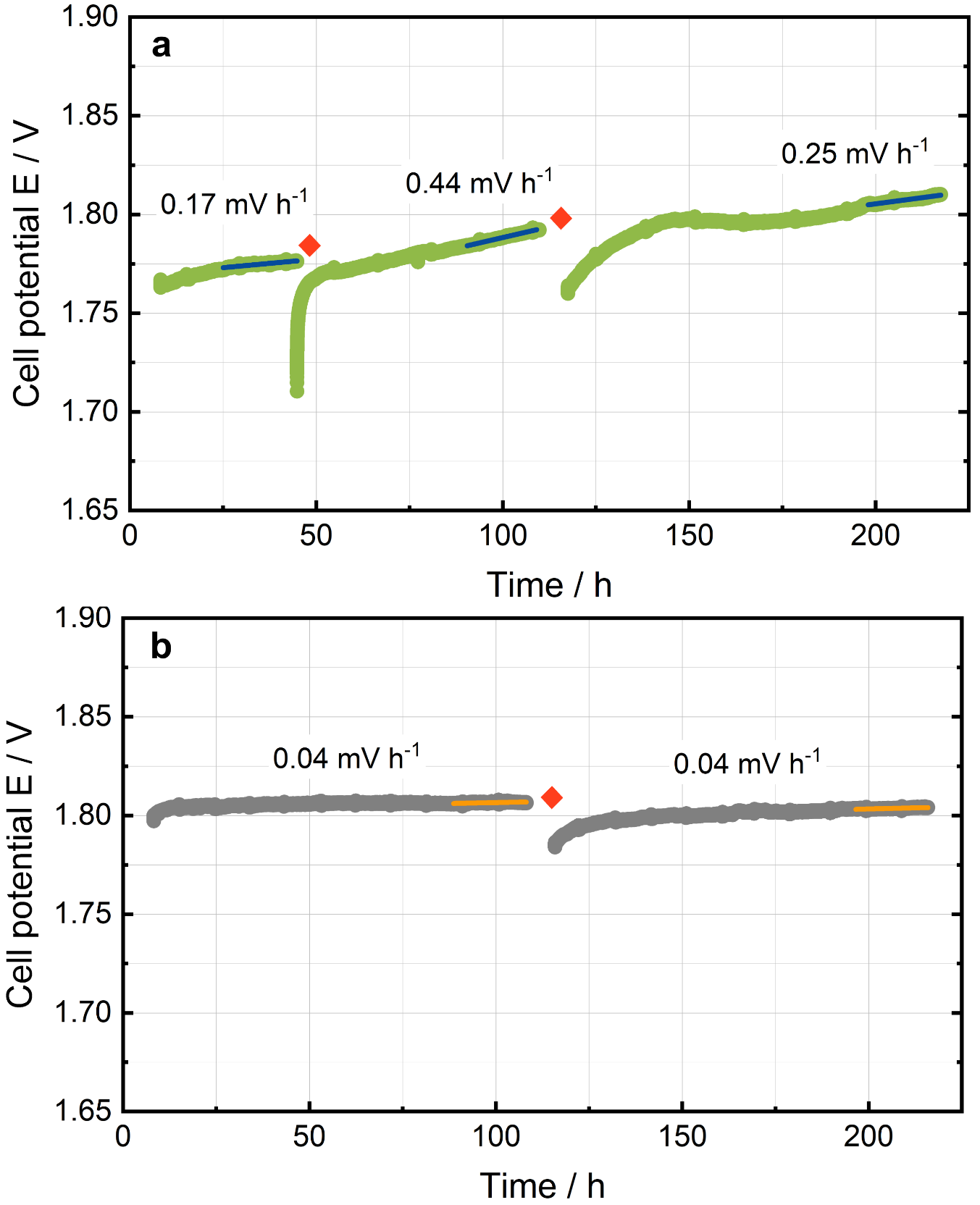


**Figure S17.** 200 h stability testing at 2 A cm^-2^ of (a) TiO_2_@IrO_x_ core-shell catalyst with 0.23 mg_Ir_ cm^-2^ and (b) Umicore Elyst Ir75 catalyst with 0.32 mg_Ir_ cm^-2^. Nafion212 membranes (~ 51 µm), a cathode loading of 0.20 mg_Pt_ cm^-2^, and platinum-coated Ti-fiber PTLs on the anode were used. Measurements were performed at 80 °C and ambient pressure while the anode was flushed with 100 ml min^-1^ DI water. Degradation rates are determined by a linear regression of the voltage increase during the last 20 h of each constant current hold.

The contributions to the performance decay can be further analyzed with the polarization curve and electrochemical impedance spectroscopy (EIS) data at the beginning of the 200 h current hold (BOT) and end of test (EOT). The potential observed at the 2 A cm^-^² step of the polarization curve increases by 40.4 mV. Using the EIS data, the contribution from the HFR increase over the 200 h hold can be quantified and compared to the overall overpotential increase. As displayed in **Figure S18**a, the overall contribution to the cell potential increase is dominated by an increase in HFR-free potential (25.5 mV), hinting toward a change in activity. A smaller contribution of about 14.9 mV stems from an increase in HFR. As comparison, the overall degradation derived from the potential increase in Figure S17 is also shown in Figure S18a (right bar). A deviation of ~ 7 mV of the cell potential increase derived from the polarization curve data compared to the current hold can be seen, hinting toward reversible cell degradation effect occuring during the long constant current hold, but the origin needs further investigation.


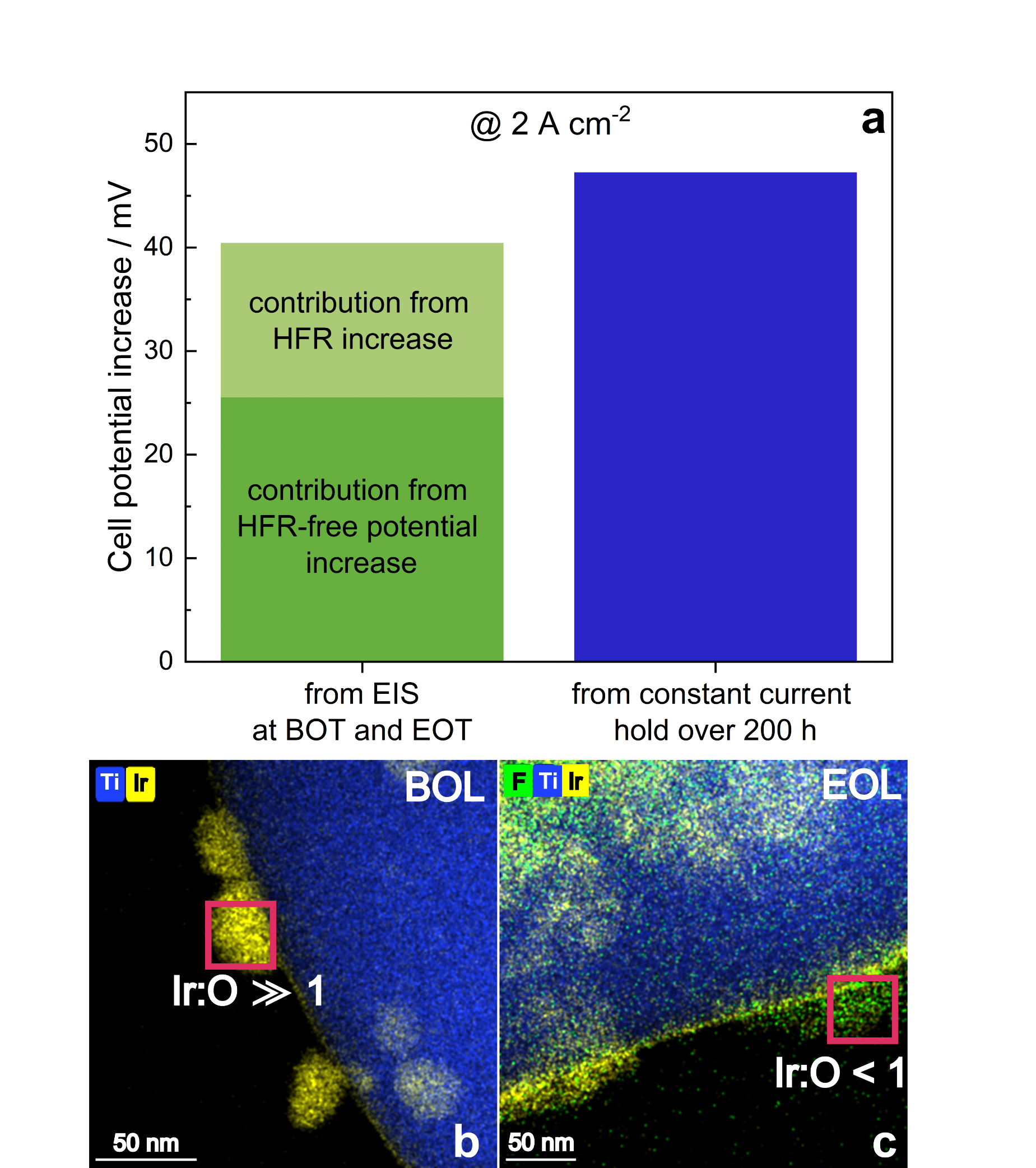


**Figure S18.** (a) Analysis of the cell potential increase from the stability test at 2 A cm^‑2^ over 200 h (cf. Figure S17). Data was calculated from electrochemical impedance spectroscopy data at the beginning of test (BOT) and end of test (EOT) at 2 A cm^‑2^ (left bar) or from potential increase measured for the constant current hold (right bar). (b), (c) STEM-EDXS images with calculated iridium-to-oxygen ratio at (b) beginning of test (BOT) and (c) end of test (EOT). Note that for BOT, the catalyst powder as prepared was measured and at EOT, the catalyst with ionomer from the tested electrode was scratched off for the measurement. Hence, at EOT, the oxygen contribution from the ionomer was subtracted for the calculation.

To investigate if a disruption of the core-shell structure causes the decrease in performance, HAADF-STEM and EDXS measurements were performed at EOT and are shown in **Figure S19.** As can be seen, the IrO_x_ shell and additional particles are unchanged after stability testing. Interestingly, calculations based on the STEM-EDXS measurements before and after 200 h at 2 A cm^‑2^ (Figure S18b and c) reveal a change in the iridium-to-oxygen ratio from BOT to EOT. At BOT, the ratio is much larger than one (Ir:O ≫ 1) and changes to smaller than one at EOT (Ir:O < 1). This further oxidation of the catalyst material throughout the testing time could be linked to the increased HFR-free potential. Note that the catalyst surface can already be changed throughout the CL fabrication. This difference is not captured here. Theoretically, a decrease in performance could additionally originate from a change in crystallinity of the iridium^[25]^, which cannot be determined from STEM-EDXS. Another possible source is interfacial effects between Pt-coated PTL and CL where a different degree of Pt oxidation possibly depends on the material in contact (i.e., amorphous or rutile iridium oxide). However, further investigation is needed to determine the complex interplay of degradation mechanisms.

For the reference catalyst, the degradation rate in the two regions before and after interruption is 0.04 mV h^-1^. In general, the degradation rate for the reference catalyst is smaller than that of the core-shell catalyst, leading to a superior performance of the reference after roughly 190 h of testing. A possible explanation is the difference in crystallinity where it was shown that crystalline IrO_2_ is more stable than amorphous IrO_x_.^[4,25]^ For more insights into the difference in degradation, further analysis is needed.

To bring the results into context, they are compared to stability tests from literature with low-loaded anodes in the following. A long-term test over 150 h at 2 A cm^-2^ conducted with similar core-shell TiO_2_@IrO_x_ particles developed in our group resulted in a degradation rate of around 0.49 mV h^-1^.^[26]^ The implementation of an iridium-nanofiber interlayer by Hegge *et al.* enabled an improvement in degradation rate from 0.7 mV h^-1^ to 0.2 mV h^-1^ over 150 h.^[27]^ It becomes clear that our particles are already stable compared to this result but could probably profit from implementing an interlayer stability-wise. Möckl *et al.* reach a superior degradation rate within the first 1000 h of 0.06 to 0.08 mV h^-1^ and even see an improvement of performance after 1000 h until 3700 h.^[25]^

Comparing these results to the 2026 target for PGM content and stability given by the U.S Department of Energy (DOE), two points need to be remarked. With an anode and cathode loading of around 0.2 mg_PGM_ cm^-2^, respectively, our experiments already stay below the target of the total PGM content (both electrodes) of 0.5 mg cm^-2^. In terms of stability, however, the degradation target of 2.3 µV h^-1^ over 1000 h is exceeded.^[28]^ It needs to be noted that for a direct comparison of the two values, a measurement over 1000 h needs to be conducted since previous publications showed that the first hours of long‑term testing are marked by a higher degradation rate. It was shown that the degradation slows down after several hundreds of hours of operation.^[24,25]^ However, such extensive long-term testing is not in the scope of this work.

In summary, our TiO_2_@IrO_x_ core-shell catalyst already exhibits good stability in a constant current operation, which is comparable to literature values. However, literature and the DOE target also show that a further improvement of the stability is possible and needed for large-scale implementation.


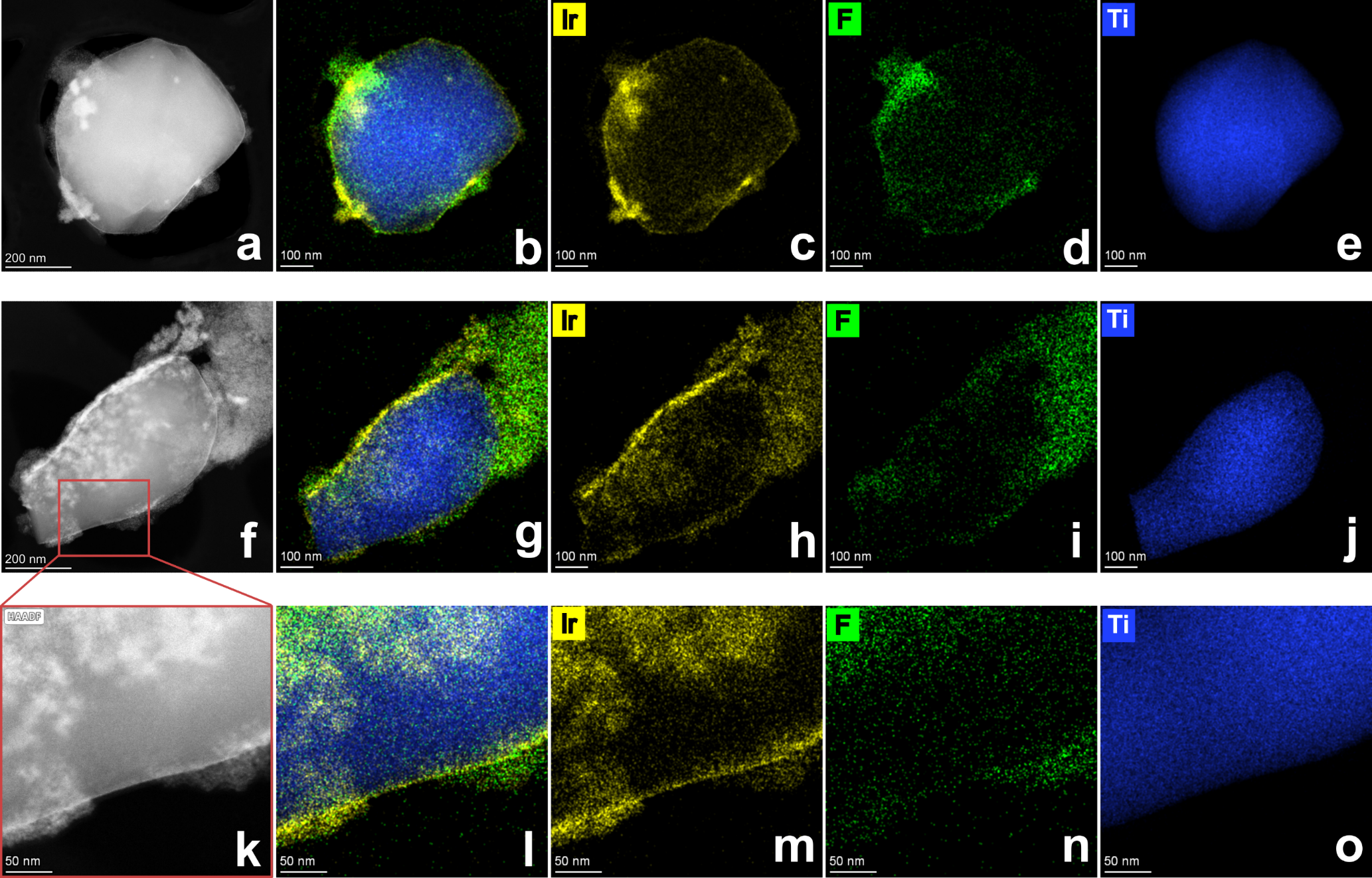


**Figure S19.** TiO_2_@IrO_x_ core-shell catalyst particles after 200 h operation at 2 A cm-2 (a), (f), (k) HAADF-STEM. (b) – (e), (g) – (j), (l) – (o) STEM-EDXS analysis of iridium (Ir), fluorine (F), and titanium (Ti) overlayed and single elements.

**3. Tables**

**Table S1.** XPS fitting parameters.

| Component | Line shape | FWHM / eV | Binding energy (BE) / eV | |
| --- | --- | --- | --- | --- |
|  |  |  | 4f_7/2_ | 4f_5/2_ |
| Ir (0) | LF(0.6,1,150,300) | 1.1 ± 0.2 | 60.9 ± 0.1 | BE(4f_7/2_) + 3 eV |
| Ir (IV) | LF(0.5,1,65,100) | Same as Ir(0) | 61.75 ± 0.1 | BE(4f_7/2_) + 3 eV |
| Ir (III) | LF(0.5,1,65,100) | Same as Ir(0) | 62.4 ± 0.1 | BE(4f_7/2_) + 3 eV |

**Table S2.** Peak area fractions of the different iridium oxidation states as determined from XPS deconvolution fitting.

|  | After photodeposition (only seeds) | After photodeposition + annealing (only shell) | After photodeposition + IPA reduction + annealing |
| --- | --- | --- | --- |
| Figure with spectrum | Figure S8 | Figure 3g | Figure 3f |
| Ir (0) | 3% | 9% | 36% |
| Ir (IV) | 48% | 73% | 53% |
| Ir (III) | 49% | 18% | 11% |

**Table S3.** Overview of mass activities of iridium-based catalysts from half-cell measurements in recent publications sorted by most recent publication date.

| **Catalyst material** | **Ir content in catalyst / wt%** | **Mass activity (A g_Ir_^-1^)** | | | **Remark** | **Reference** |
| --- | --- | --- | --- | --- | --- | --- |
|  |  | **at 1.5 V vs. RHE** | **at 1.55 V vs. RHE** | **at 1.6 V vs. RHE** |  |  |
| **TiO_2_@IrO_x_** | **40** | **4.7 ± 0.7** | **49 ± 6** | **236 ± 13** |  | **This work** |
| **IrO_x_ (Alfa Aesar)** | **84.5** | **17.0 ± 0.6** | **139 ± 6** | **530 ± 50** |  | **This work** |
| **IrO_2_/TiO_2_** | **75** | **0.90 ± 0.11** | **10.6 ± 0.6** | **65 ± 1** |  | **This work** |
| Ir black | 100 |  | 557.9 ± 28.5 |  |  | Baik *et al.*^[29]^ |
| Ir/C | 30 |  | 445.9 ± 7.3 |  |  | Baik *et al.*^[29]^ |
| Ir/mesoporous Ta_2_O_5_ | 30 |  | 876.1 ± 125.1 |  |  | Baik *et al.*^[29]^ |
| Ir/TiO_2_ | 30 |  | 209.0 ± 16.9 |  |  | Baik *et al.*^[29]^ |
| Ir/ATO | 15 - 65 |  | 80 - 130 |  | Series of catalysts with different Ir wt%. | Gollasch *et al.*^[30]^ |
| IrO_2_/ATO | 8 - 65 |  | 20 - 100 |  | Series of catalysts with different Ir wt%. | Gollasch *et al.*^[30]^ |
| IrO_2_/TiO_2_ *via* photodeposition | 25 | ~ 20 |  |  | Potentials plotted vs. SCE. | Dimitrova *et al.^[31]^* |
| IrOOH/TiO_2_ | 30 |  | 62 (at 1.53 V) | 1048 (at 1.65 V) |  | Böhm *et al.*^[32]^ |
| IrOOH/TiO_2_ | 45 |  | 71 (at 1.53 V) | 1047 (at 1.65 V) |  | Böhm *et al.*^[32]^ |
| Ir-Pt-TiO_2_ *via* photodeposition | 25 (+ 18 Pt) |  |  | 160 |  | Regmi *et al.*^[33]^ |
| IrO­_2_@TiO_2_ (shell@core) | 50 |  | 51 (at 1.525 V) | 364 | Measured in SFC. | Van Pham, Bühler *et al.*^[26]^ |
| IrO_x_/ATO |  | 166 |  |  | No ionomer in RDE ink. | Hartig-Weiss *et al.*^[34]^ |
| IrO_2_@Ir/TiN | 60 |  |  | 480.4 |  | Li *et al.*^[35]^ |
| Mesoporous Ir/TiO_x_ | 30 |  |  | 158.3 |  | Bernsmeier *et al.*^[36]^ |
| IrO_2_-TiO­_2_ | 56 (40 mol%) |  | 70 (at 1.525 V) |  |  | Oakton *et al* ^[37]^ |

**Table S4.** Overview of iridium-specific power densities and durability of iridium-based catalysts from full-cell measurements in recent publications sorted by most recent publication date. Only publications with loadings < 0.5 mg_Ir_ cm^‑2^ are listed.

| **Catalyst material** | **Ir content in catalyst** | **Ir loading** | **Membrane** | **Ir-specific power density** | **Durability** | | **Source** |
| --- | --- | --- | --- | --- | --- | --- | --- |
|  | wt% | mg_Ir_ cm^-2^ |  | @1.79 V / kW g_Ir_^-1^ | Testing conditions | Degradation rate / mV h^-1^ |  |
| **TiO_2_@IrO_x_** | **40** | **0.22** | **N212** | **17.90** | **200 h at 2  A cm^‑2^** | **0.25** | **This work** |
| **IrO_2_/TiO_2_ (Umicore)** | **75** | **0.3** | **N212** | **10.44** | **200 h at 2  A cm^‑2^** | **0.04** | **This work** |
| Ir/ mesoporous Ta_2_O_5_ | 30 | 0.3 | N115 | 10.98 | 48 h at 1  A cm^‑2^ | 0.36 | Baik et al. ^[29]^ |
| Hydrous IrO_x_ supported on TiO_2_ | 49.2 | 0.25 | N117 | 8.95 | 3700 h (switched every 10 min between 0.1, 1.75, and 2.0  A cm^‑2^) | 0.06 – 0.08 (within 1000 h);  - 8·10^-3^ (1000 h until 3700 h) | Möckl *et al.* ^[25]^ |
| Ir supported on TiO_2_-MoO_x_ | 26 | 0.5 | N115 | 6.77 | 50 h at 1  A cm^‑2^ | 2.2 | Kim *et al.* ^[38]^ |
| IrO_2_@TiO_2_  (shell@core) | 50 | 0.4 | N212 | 9.40 | 150 h at 2  A cm^‑2^ | 0.49 | Van Pham, Bühler *et al.* ^[26]^ |
| Combined IrO_x_ nanoparticles and IrOx nanofibers | 100 | 0.2 | N115 | 16.11 | 150 h at 2 A cm^‑2^ | 0.2 | Hegge *et al.* ^[27]^ |
| IrO_x_ | 100 | 0.08 | N117 | 42.19 (No polarization curve shown: Ir-specific power density calculated at 1.875 V) | 4500 h at 1.8  A cm^‑2^ | 0.03 | Yu *et al.* ^[39]^ |

Received: ((will be filled in by the editorial staff))
Revised: ((will be filled in by the editorial staff))
Published online: ((will be filled in by the editorial staff))

References

[1] J. Schindelin, I. Arganda-Carreras, E. Frise, V. Kaynig, M. Longair, T. Pietzsch, S. Preibisch, C. Rueden, S. Saalfeld, B. Schmid, J.-Y. Tinevez, D. J. White, V. Hartenstein, K. Eliceiri, P. Tomancak, A. Cardona, *Nat. Methods* 2012, *9*, 676.

[2] de Fang, F. He, J. Xie, L. Xue, *J. Wuhan Univ. Technol.-Mat. Sci. Edit.* 2020, *35*, 711.

[3] N. Fairley, V. Fernandez, M. Richard‐Plouet, C. Guillot-Deudon, J. Walton, E. Smith, D. Flahaut, M. Greiner, M. Biesinger, S. Tougaard, D. Morgan, J. Baltrusaitis, *Appl. Surf. Sci. Adv.* 2021, *5*, 100112.

[4] S. Geiger, O. Kasian, B. R. Shrestha, A. M. Mingers, K. J. J. Mayrhofer, S. Cherevko, *J. Electrochem. Soc.* 2016, *163*, F3132-F3138.

[5] V. Pfeifer, T. E. Jones, J. J. Velasco Vélez, C. Massué, R. Arrigo, D. Teschner, F. Girgsdies, M. Scherzer, M. T. Greiner, J. Allan, M. Hashagen, G. Weinberg, S. Piccinin, M. Hävecker, A. Knop-Gericke, R. Schlögl, *Surf. Interface Anal.* 2016, *48*, 261.

[6] S. J. Freakley, J. Ruiz‐Esquius, D. J. Morgan, *Surf. Interface Anal.* 2017, *49*, 794.

[7] N. Doebelin, R. Kleeberg, *J Appl Crystallogr* 2015, *48*, 1573.

[8] S. Gražulis, D. Chateigner, R. T. Downs, A. F. T. Yokochi, M. Quirós, L. Lutterotti, E. Manakova, J. Butkus, P. Moeck, A. Le Bail, *J Appl Crystallogr* 2009, *42*, 726.

[9] A. Fraser, Z. Zhang, G. Merle, J. Gostick, J. Barralet, *Electroanalysis* 2018, *30*, 1897.

[10] L. J. van der Pauw, *Philips Tech. Rev.* 1958, *20*, 220.

[11] F. S. Oliveira, R. B. Cipriano, F. T. Da Silva, E. C. Romão, dos Santos, C. A. M., *Sci Rep* 2020, *10*, 16379.

[12] S. Brunauer, P. H. Emmett, E. Teller, *J. Am. Chem. Soc.* 1938, *60*, 309.

[13] J. Wang, Y. Karen Chen, Q. Yuan, A. Tkachuk, C. Erdonmez, B. Hornberger, M. Feser, *Appl. Phys. Lett.* 2012, *100*.

[14] P. Gilbert, *J. Theor. Biol.* 1972, *36*, 105.

[15] W. van Aarle, W. J. Palenstijn, J. de Beenhouwer, T. Altantzis, S. Bals, K. J. Batenburg, J. Sijbers, *Ultramicroscopy* 2015, *157*, 35.

[16] O. Kasian, S. Geiger, K. J. J. Mayrhofer, S. Cherevko, *Chem. Rec.* 2019, *19*, 2130.

[17] S. O. Klemm, A. A. Topalov, C. A. Laska, K. J. Mayrhofer, *Electrochem. Commun.* 2011, *13*, 1533.

[18] J. Landesfeind, J. Hattendorff, A. Ehrl, W. A. Wall, H. A. Gasteiger, *J. Electrochem. Soc.* 2016, *163*, A1373-A1387.

[19] R. Makharia, M. F. Mathias, D. R. Baker, *J. Electrochem. Soc.* 2005, *152*, A970.

[20] T. Ioroi, T. Nagai, Z. Siroma, K. Yasuda, *Int. J. Hydrog. Energy* 2022, *47*, 38506.

[21] S. Geiger, O. Kasian, M. Ledendecker, E. Pizzutilo, A. M. Mingers, W. T. Fu, O. Diaz-Morales, Z. Li, T. Oellers, L. Fruchter, A. Ludwig, K. J. J. Mayrhofer, M. T. M. Koper, S. Cherevko, *Nat Catal* 2018, *1*, 508.

[22] J. Knöppel, M. Möckl, D. Escalera-López, K. Stojanovski, M. Bierling, T. Böhm, S. Thiele, M. Rzepka, S. Cherevko, *Nat Commun* 2021, *12*, 2231.

[23] M. Milosevic, T. Böhm, A. Körner, M. Bierling, L. Winkelmann, K. Ehelebe, A. Hutzler, M. Suermann, S. Thiele, S. Cherevko, *ACS Energy Lett.* 2023, *8*, 2682.

[24] A. Weiß, A. Siebel, M. Bernt, T.-H. Shen, V. Tileli, H. A. Gasteiger, *J. Electrochem. Soc.* 2019, *166*, F487-F497.

[25] M. Möckl, M. F. Ernst, M. Kornherr, F. Allebrod, M. Bernt, J. Byrknes, C. Eickes, C. Gebauer, A. Moskovtseva, H. A. Gasteiger, *J. Electrochem. Soc.* 2022, *169*, 64505.

[26] C. van Pham, M. Bühler, J. Knöppel, M. Bierling, D. Seeberger, D. Escalera-López, K. J. Mayrhofer, S. Cherevko, S. Thiele, *Appl. Catal. B: Environ.* 2020, *269*, 118762.

[27] F. Hegge, F. Lombeck, E. Cruz Ortiz, L. Bohn, M. von Holst, M. Kroschel, J. Hübner, M. Breitwieser, P. Strasser, S. Vierrath, *ACS Appl. Energy Mater.* 2020, *3*, 8276.

[28] Energy.gov, *Technical Targets for Proton Exchange Membrane Electrolysis* 2023, <https://www.energy.gov/eere/fuelcells/technical-targets-proton-exchange-membrane-electrolysis>, accessed: June, 2024.

[29] C. Baik, J. Cho, J. in Cha, Y. Cho, S. S. Jang, C. Pak, *Journal of Power Sources* 2023, *575*, 233174.

[30] M. Gollasch, J. Schmeling, C. Harms, M. Wark, *Adv. Mater. Interfaces* 2023, *10*, 2300036.

[31] N. Dimitrova, A. Banti, O.-N. Spyridou, A. Papaderakis, J. Georgieva, S. Sotiropoulos, E. Valova, S. Armyanov, D. Tatchev, A. Hubin, K. Baert, *J. Electroanal. Chem.* 2021, *900*, 115720.

[32] D. Böhm, M. Beetz, C. Gebauer, M. Bernt, J. Schröter, M. Kornherr, F. Zoller, T. Bein, D. Fattakhova-Rohlfing, *Appl. Mater. Today* 2021, *24*, 101134.

[33] Y. N. Regmi, E. Tzanetopoulos, G. Zeng, X. Peng, D. I. Kushner, T. A. Kistler, L. A. King, N. Danilovic, *ACS Catal.* 2020, *10*, 13125.

[34] A. Hartig-Weiss, M. Miller, H. Beyer, A. Schmitt, A. Siebel, A. T. S. Freiberg, H. A. Gasteiger, H. A. El-Sayed, *ACS Appl. Nano Mater.* 2020, *3*, 2185.

[35] G. Li, K. Li, L. Yang, J. Chang, R. Ma, Z. Wu, J. Ge, C. Liu, W. Xing, *ACS Appl. Mater. Interfaces* 2018, *10*, 38117.

[36] D. Bernsmeier, M. Bernicke, R. Schmack, R. Sachse, B. Paul, A. Bergmann, P. Strasser, E. Ortel, R. Kraehnert, *ChemSusChem* 2018, *11*, 2367.

[37] E. Oakton, D. Lebedev, M. Povia, D. F. Abbott, E. Fabbri, A. Fedorov, M. Nachtegaal, C. Copéret, T. J. Schmidt, *ACS Catal.* 2017, *7*, 2346.

[38] E.-J. Kim, J. Shin, J. Bak, S. J. Lee, K. h. Kim, D. Song, J. Roh, Y. Lee, H. Kim, K.-S. Lee, E. Cho, *Appl. Catal. B: Environ.* 2021, *280*, 119433.

[39] H. Yu, L. Bonville, J. Jankovic, R. Maric, *Applied Catalysis B: Environmental* 2020, *260*, 118194.
